# Probing the potential of CnaB-type domains for the design of tag/catcher systems

**DOI:** 10.1371/journal.pone.0179740

**Published:** 2017-06-27

**Authors:** Marlene Pröschel, Max E. Kraner, Anselm H. C. Horn, Lena Schäfer, Uwe Sonnewald, Heinrich Sticht

**Affiliations:** 1 Lehrstuhl für Biochemie, Department Biologie, Friedrich-Alexander-Universität Erlangen-Nürnberg, Erlangen, Germany; 2 Bioinformatik, Institut für Biochemie, Friedrich-Alexander-Universität Erlangen-Nürnberg, Erlangen, Germany; University of Queensland, AUSTRALIA

## Abstract

Building proteins into larger, post-translational assemblies in a defined and stable way is still a challenging task. A promising approach relies on so-called tag/catcher systems that are fused to the proteins of interest and allow a durable linkage via covalent intermolecular bonds. Tags and catchers are generated by splitting protein domains that contain intramolecular isopeptide or ester bonds that form autocatalytically under physiological conditions. There are already numerous biotechnological and medical applications that demonstrate the usefulness of covalent linkages mediated by these systems. Additional covalent tag/catcher systems would allow creating more complex and ultra-stable protein architectures and networks. Two of the presently available tag/catcher systems were derived from closely related CnaB-domains of *Streptococcus pyogenes* and *Streptococcus dysgalactiae* proteins. However, it is unclear whether domain splitting is generally tolerated within the CnaB-family or only by a small subset of these domains. To address this point, we have selected a set of four CnaB domains of low sequence similarity and characterized the resulting tag/catcher systems by computational and experimental methods. Experimental testing for intermolecular isopeptide bond formation demonstrated two of the four systems to be functional. For these two systems length and sequence variations of the peptide tags were investigated revealing only a relatively small effect on the efficiency of the reaction. Our study suggests that splitting into tag and catcher moieties is tolerated by a significant portion of the naturally occurring CnaB-domains, thus providing a large reservoir for the design of novel tag/catcher systems.

## Introduction

In synthetic biology and biotechnology, artificial linkage of proteins or peptides plays a crucial role in the formation of stable and complex protein architectures. These protein architectures offer a wide range of applications, including for instance efficiency enhancement of metabolic pathways by creating multi-enzyme complexes that facilitate substrate channeling [1]. However, the design of post-translational multiprotein assemblies is often hampered by weak and reversible protein-protein or protein-peptide interactions [2, 3]. Furthermore, linkage of several proteins into one functional complex is often limited by the restricted number of comparably efficient and specific interaction domains. To avoid the risk of dissociation and to allow formation of stable protein complexes, there is a need to develop a toolbox of specific and covalent protein connectors.

In living systems—eukaryotes as well as prokaryotes—covalent and non-covalent linkages between and within polypeptides are highly relevant to control protein activity as well as stability and help to organize and regulate metabolic flux or signal transduction pathways [4–8]. Non-covalent linkages are mainly stabilized by hydrophobic and ionic interactions as well as hydrogen bonds between specific protein interaction domains [7]. As for instance nicely demonstrated by a metabolic engineering approach from Dueber et al. [1], interactions between well-known and widespread adaptor domains (e.g. SH3, PDZ) and their cognate peptide ligands are frequently used as scaffolds to create programmable, higher-order protein assemblies [9]. Although being highly specific, these reversible interactions do not resist mechanical forces and dissociate over time. Therefore, applications of protein complexes based on non-covalent protein-protein or peptide-protein interactions are restricted to mild conditions and cannot be used when exposed to higher forces. Alternatives to non-covalent interactions are covalent linkages.

Covalent crosslinks in proteins best known from nature include redox-sensitive, reversible disulfide bonds between two cysteine residues, but also comprise ester [10] and thioester bonds [11–14] as well as irreversible isopeptide bonds [7, 15] between an amino and a carboxylate or carbamide group, of which at least one belongs to the protein side chain. Such peptide bonds can thus either connect the side chains of lysine to aspartate or asparagine, respectively, or the side chains of those residues to the terminus of the main chain.

Intermolecularly, such isopeptide bonds can link two proteins or subunits, with enzyme-mediated and ATP-dependent ubiquitylation, sumoylation and transglutamination as most prominent examples [3, 7]. In gram-positive bacteria, intermolecular isopeptide bonds play a key role in pilus formation. Bacterial pili are filamentous structures that extend from the bacterial cell surface and mediate, inter alia, host cell adhesion [7]. Pilus subunits are assembled by several transpeptidase enzymes called sortases that covalently link the different subunits by isopeptide bonds [15, 16].

Besides intermolecular isopeptide bonds, intramolecular isopeptide bonds are widespread in in immunoglobulin-like domains of cell-surface proteins from gram-positive bacteria [3, 7]. The first intramolecular isopeptide bond was identified within the major backbone pilin subunit Spy0128 from the human pathogen *Streptococcus pyogenes* [15] and subsequent studies revealed related structures in other bacteria [7, 17]. The isopeptide bonds within these domains form during protein folding via an autocatalytic mechanism mediated by a catalytic aspartate or glutamate residue, enforced proximity of the reactive amino acids (lysine and either asparagine or aspartate), and a hydrophobic protein environment often including aromatic residues [7]. This intramolecular lock confers enhanced conformational, mechanical, proteolytic, thermal, and pH stability to their parent protein structures [3, 6, 13] and therefore makes it attractive for a wide spectrum of applications in synthetic biology and biotechnology [3].

**Fig 1:**
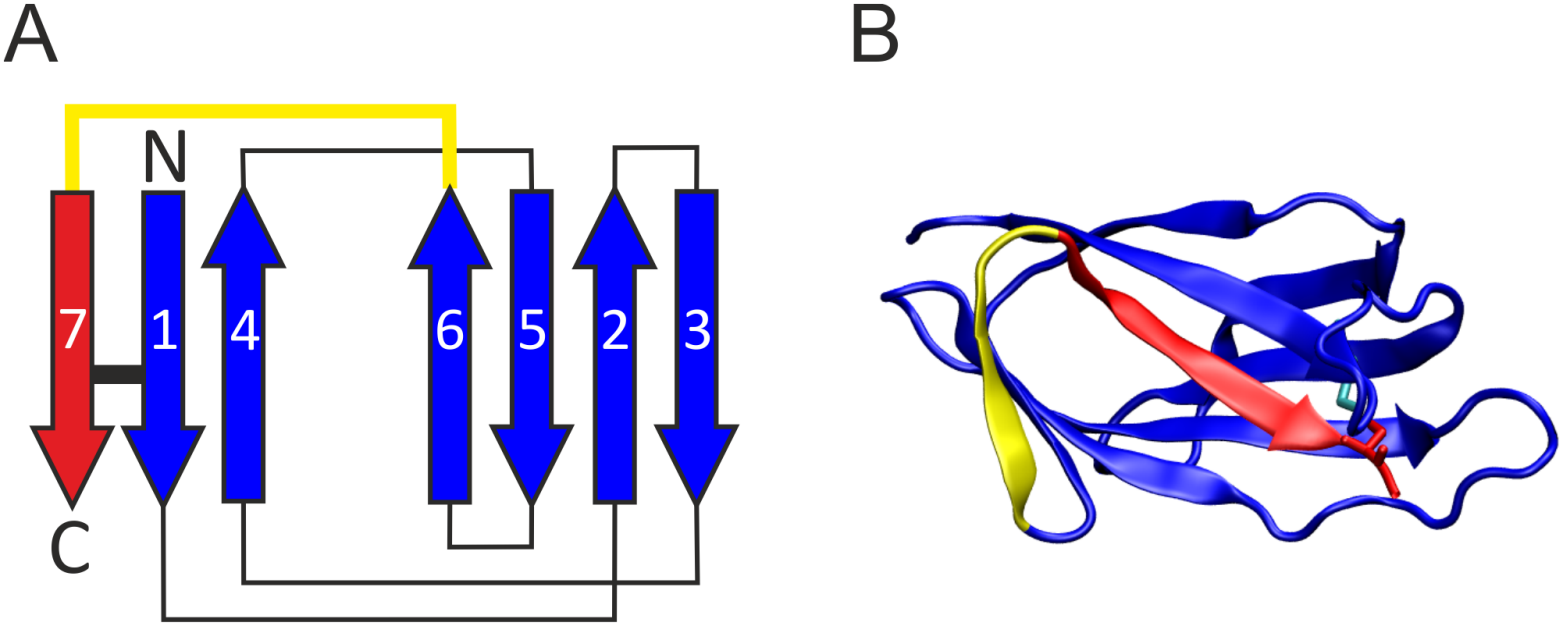
Structural features of CnaB domains and derived split-protein systems. (A) Topology diagram of the CnaB fold. The β-strands are depicted as arrows and a black bar marks the locations of the isopeptide bond. The color coding indicates the design concept for CnaB-based split-protein systems. In these systems, the yellow sequence stretch is removed, resulting in a catcher (blue) and tag (red) moiety. The isopeptide bond is retained in this split-protein system and forms after association of the tag and catcher parts. (B) Structure of the CnaB domain from the *S*. *pyogenes* fibronectin binding FbaB (pdb code: 2x5p[54]) that served as template for the design of the SpyTag/SpyCatcher system (color coding as in (A)). The Asp and Lys that form the isopeptide bond are shown as sticks and colored in red and cyan, respectively.

The idea to use a protein exhibiting an intramolecular isopeptide bond for engineered, intermolecular protein-protein linkages was pioneered in the group of Mark Howarth by generating the first two tag/catcher systems based on the backbone pilin Spy0128 [18]. For that purpose either the N-terminal or the C-terminal domain of Spy0128 was split into two parts to allow for the formation of an intermolecular isopeptide bond [18]. Another split-protein system was designed by this group based on the CnaB domain of the fibronectin-binding protein FbaB from *Streptococcus pyogenes* [19]. The CnaB fold comprises seven conserved β-strands forming a sandwich of a three-stranded and four-stranded β-sheet. In this fold, the first and last β-strand pair in a parallel fashion and are connected by an intramolecular isopeptide bond. (Fig 1A). Within the CnaB domain of FbaB this isopeptide bond forms spontaneously between a reactive lysine and a reactive aspartate (Fig 1B). A neighboring glutamate, responsible for proton shuffling and therefore stabilizing the intermediate state, catalyzes the isopeptide bond formation. By rational splitting of this CnaB domain and extensive optimization of the resulting parts, two reactive, genetically encoded protein partners—SpyTag and SpyCatcher—were developed [19]. Two proteins fused to SpyTag and SpyCatcher, respectively, can then be covalently linked by intermolecular isopeptide bond formation simply upon mixing the reactants. With the advent of this covalent split-protein system many applications have been demonstrated [20], which benefit from the system´s robustness and independence from most experimental conditions [21], e.g. cell surface labeling in fluorescence microscopy [18], enzyme resilience to boiling [22–25], bioactive hydrogels [26], and protein assembly [21, 27–32].

To allow the design of more complex and programmable multi-protein complexes, additional tag/catcher systems have been engineered: The SnoopTag/SnoopCatcher pair, which also relies on the formation of an intermolecular isopeptide bond, was derived from the D4 Ig-like domain of the tip-associated adhesin RrgA from *Streptococcus pneumoniae* [33]. In contrast to the Spy-system, this domain was split after the first N-terminal β-strand. The resulting system contains a lysine in the tag and an asparagine in the catcher thus avoiding cross-reactivity with the Spy-system [33]. Very recently, it has also been demonstrated that not only isopeptide bonds, but also ester bonds can be exploited to generate tag/catcher systems [34]. As a difference compared to the isopeptide bond, ester bond formation is reversible under certain conditions allowing both rational assembling and disassembling of complex protein nanomaterials [34]. Another tag/catcher system (SdyTag/SdyCatcher) has been derived from a CnaB-domain of a *Streptococcus dysgalactiae* fibronectin-binding protein, which exhibits 63% sequence identity to the CnaB-domain template of the Spy-system [35].

It is tempting to speculate that cell-surface proteins from the CnaB-family, which already provided the template for the design of the Spy- and Sdy-systems, represent a promising source of additional tag/catcher systems. The CnaB-domains are a large and divergent protein family consisting of 1719 sequences from 275 species (according to PFAM entry PF05738, PFAM Version 31.0) [9]. It is yet unclear whether domain splitting is generally tolerated within CnaB-domains or only by a small subset of closely related CnaB domains.

To address this point we established a versatile screening protocol that allows assessing CnaB-domains as candidates for the design of tag/catcher systems. We have exemplarily selected four CnaB domains of low sequence similarity and characterized the resulting tag/catcher systems by computational and experimental methods. Our study reveals that two of these systems retain the ability of their parent systems to form isopeptide bonds. Thus, we conclude that a significant portion of the large family of CnaB-type domains can be used as templates for the design of novel tag/catcher systems in future.

## Materials and methods

### System selection and molecular dynamics simulations

**Fig 2:**
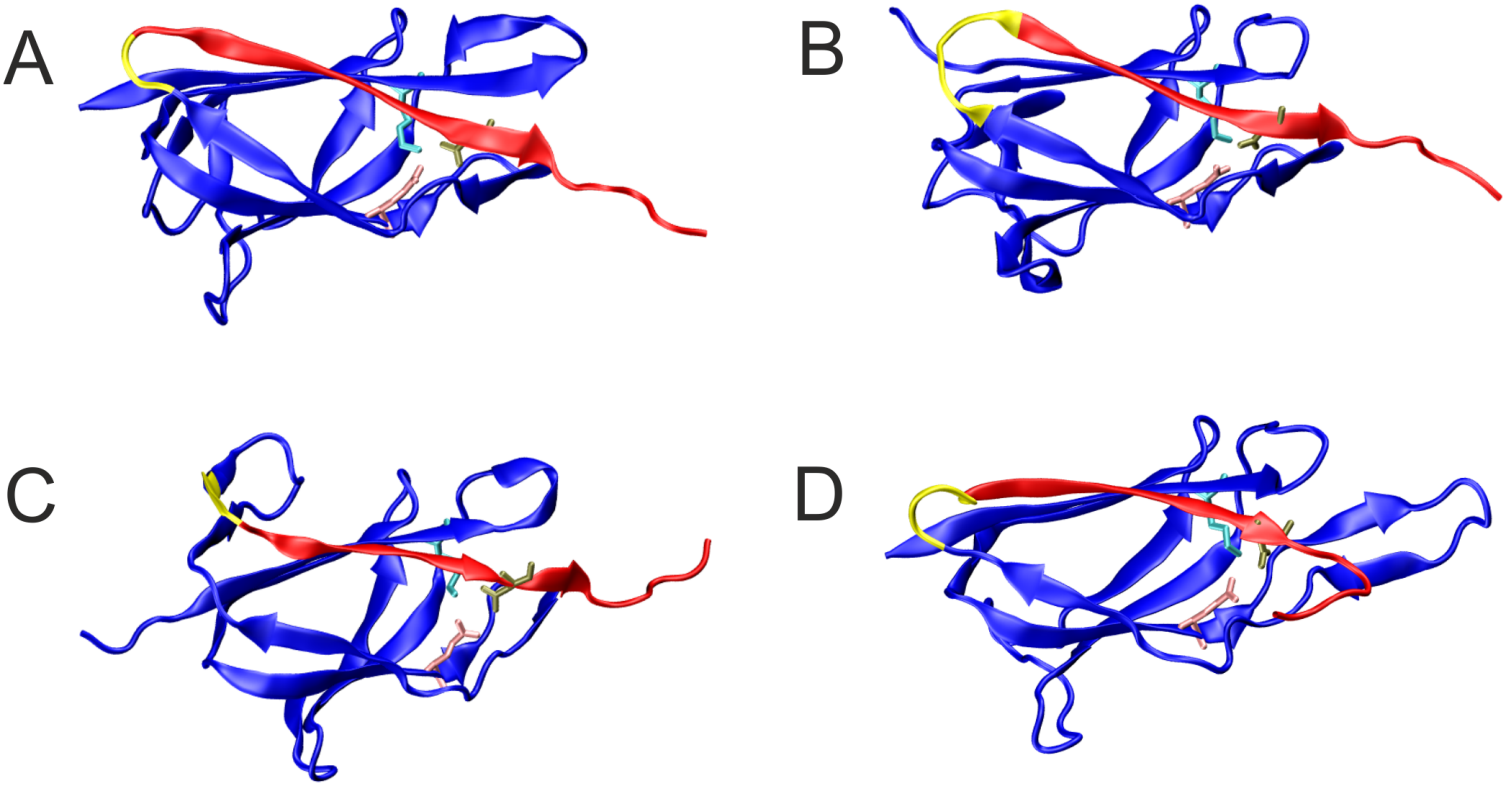
Structures of the CnaB-type domains that served as starting point for the design of the tag/catcher systems. The systems are named according to the PDB entry they were derived from (see Table 1 for details) (A) 3phs; (B) 4oq1; (C) 3kptN; (D) 3kptC. Residues of the tag, catcher, and deleted regions are colored red, blue, and yellow, respectively (see Table 1 for details on the domain boundaries); active-site residues Asn, Lys and Glu are shown as sticks.

Candidate CnaB-domains were selected according to the following criteria: (i) low (<30%) sequence identity between the candidates and also to the original Spy-system (ii) existence of an experimental structure confirming the presence of an isopeptide bond in the intact domain. Based on these criteria, four CnaB domains from three different proteins were selected and regions for tag and catcher constructs were defined by a visual inspection of the structures (Table 1, Fig 2). For molecular dynamics (MD) simulations, the original N- and C-termini of the CnaB domain were capped with acetyl or N-methyl groups, while the newly created termini were kept ionic. The tag/catcher complexes were simulated without an isopeptide bond to mimic the initial stage of recognition, i.e. where the tag peptide chain binds the catcher region in the reaction-competent conformation. To assess the effect of cleavage in the tag/catcher-systems control simulations were performed for the intact parent domains of each system. Apart from the absence of the linking residues, simulation setup for tag/catcher and domain systems was identical.

**Table 1:** Overview of tag/catcher systems designed in the present study. The names of the systems were derived from the PDB code of the respective crystal structure. 3kptN and 3kptC indicate that these constructs have been derived from the N- and C-terminal domain of PDB entry 3KPT, respectively.

System	3phs	4oq1	3kptN	3kptC
Organism	*Streptococcus agalactiae*	*Streptococcus pneumoniae*	*Bacillus cereus*	*Bacillus cereus*
Protein	Minor basal pilin GBS52	Ancillary basal pilin RrgC	Major backbone pilin BcpA	Major backbone pilin BcpA
Domain	N2	D2	CNA2	CNA3
PDB code	3PHS [53]	4OQ1 [17]	3KPT [49]	3KPT [49]
Residues catcher/tag ^a^	152-245/248-263	146-240/246-259	164-255/258-272	409-500/505-518 ^b^

^a^ The domain sequence starts from the first catcher residue, includes the deleted residues, and extends to the last tag residue.

^b^ The three C-terminal residues of the tag peptide (Pro-Thr-Lys) are an artificial extension derived from the SpyTag system

Monovalent counter ions were added for electrical neutralization, and then all systems were immersed in a truncated octahedral box of TIP3P [36] water with a minimum distance of 10 Å to the border. In a three-step minimization with decreasing harmonic restraints all systems were relaxed. During the following equilibration, the systems were heated up to 310 K in three steps. Firstly, the protein atoms were restrained at their initial positions with a force constant of 5 kcal mol^-1^Å^-2^ for 0.1 ns; secondly, only the backbone carbon alpha atoms were restrained for 0.4 ns; and in a final 0.5 ns phase, all restraints were removed. All systems were subsequently simulated for 200 ns under constant-pressure, constant-temperature (NPT) conditions.

All MD simulations were performed with the AMBER suite [37] using the ff99SB force field [38, 39] and default program settings according to established protocols [40, 41]. Periodic boundary conditions were applied and a time step of 2 fs was chosen because of the SHAKE algorithm. For editing and visualization purposes Sybyl 7.3 [42] and VMD [43] were used, respectively. MD trajectories were analyzed with cpptraj [44]. For the analysis of the structural stability, a system-specific core region comprising the more rigid secondary structure elements was used containing residues 167–207, 183–223, 186–225, and 429–469 for 3phs, 4oq1, 3kptN, and 3kptC, respectively. These regions were used in the analysis of the root mean square deviation (RMSD) from the initial structure.

### Gene synthesis and cloning

Synthetic catcher and tag constructs were ordered at Thermo Fisher Scientific (GeneArt Strings DNA Fragments). The DNA-sequences of these constructs were used as obtained from bioinformatics analysis with codon optimization for *Escherichia coli* expression. In addition a hemagglutinin (HA)-tag was added to the catcher constructs. The short tag sequences were C-terminally fused to maltose binding protein from *E*. *coli* (malE gene without signal peptide) separated by a linear glycine serine linker (GS linker: GSGSGSG). All constructs (for sequences see S1 Text) contain BamHI/SalI restriction sites for directed cloning into the pQE-9 destination vector for bacterial expression (Qiagen; QIAexpress pQE vectors [45].

After creating compatible sticky ends by BamHI (Thermo Fisher Scientific) and SalI (Thermo Fisher Scientific) digestion and purification using the PCR Purification Kit from Qiagen, the catcher and tag constructs were ligated into BamHI/SalI digested pQE-9 bacterial expression vector using T4 DNA ligase (Thermo Fisher Scientific). Subsequent transformation into XL1Blue chemically competent *E*. *coli* cells and plasmid isolation were performed. The sequences of all pQE-9 expression constructs were verified using Sanger Sequencing (GATC Biotech).

The fusion constructs, which consisted of mCherry and catcher connected via the GS linker (mCherry-catcher) were cloned using overlap-extension PCR. mCherry is a red monomer derived from mRFP1 [46] that is an alternative fluorescent fusion protein to eGFP, which has been used as a fusion partner in previous tag/catcher systems [35]. A fluorescent protein was used as fusion partner because it allows easy visual detection when it is expressed and purified. Moreover mCherry is well expressed as soluble protein in *E*. *coli* cells. High-fidelity Phusion-DNA Polymerase (Thermo Fisher Scientific) was used for amplification. The cloning scheme and primers used (primers 1–13) are listed in S1 and S2 Tables. Final overlap PCR products were purified using gel extraction according to the manufacturer´s protocol (Qiagen Gel Extraction Kit). BamHI and SalI digestion and subsequent purification of the digested inserts following the PCR Purification Kit from Qiagen was accompanied by T4 DNA ligation into similarly digested pQE-9 vector. Constructs were transformed into chemically competent *E*. *coli* XL1Blue cells and sequences were verified by Sanger sequencing.

4oq1 and 3kptC peptide tag variants suggested from *in silico* analysis were cloned (for sequences see S1 Text). Directed mutagenesis was performed by standard PCR methods using appropriate primers (S1 Table: primers 14–21; cloning schemes in S3 and S4 Tables) and templates. Phusion-DNA Polymerase was used for amplification. Final PCR products were extracted from agarose gel (1xTBE buffer) following the manufacturer´s instructions (Qiagen Gel Extraction Kit). BamHI/SalI digestion was followed by another purification step (PCR Purification Kit, Qiagen) and T4 ligation into the bacterial expression vector pQE-9 that was previously BamHI/SalI digested and purified. Sequences were verified by Sanger sequencing.

Six mutants (N252A, K155A, E222Q for 4oq1 and N512A, K417A, E472Q for 3kptC), in which one residue of the active site was mutated, were generated either by standard PCR mutagenesis using Phusion-DNA Polymerase and mutation containing primers (S1 Table: primers 22–35) or by Gibson assembly (Gibson Assembly Cloning Kit, New England Biolabs) when the mutation site was internal (Gibson assembly for 3kptC^C^ E472Q) (cloning schemes are presented in S5 and S6 Tables). For the classical cloning procedure using compatible sticky ends to ligate the insert into the destination vector (pQE-9), BamHI/SalI digest of the gel-extracted PCR-products was performed prior to ligation into similarly digested pQE-9 vector. Transformation into *E*. *coli* XL1Blue cells, plasmid isolation and Sanger sequencing verified the correct mutants. For the Gibson cloning the PCR products were extracted from agarose gel and manufacturer´s instructions were followed when performing Gibson reaction using 2xGibson Assembly Master Mix (NEB). Transformation into *E*. *coli* XL1Blue cells, plasmid isolation and Sanger sequencing were performed.

### Recombinant protein expression and purification

The pQE-9 expression constructs containing the gene of interest N-terminally fused to the His_6_ tag encoded in the pQE-9 vector, were transformed into chemically competent M15 [pREP4] *E*. *coli* cells (Qiagen) and plated on LB agar plates with 200 μg/ml ampicillin and 25 μg/ml kanamycin.

Single colonies were grown overnight at 28°C in liquid LB medium (10g NaCl, 10g Trypton, 5g yeast extract per 1 liter) containing 25 μg/ml kanamycin and 200 μg/ml ampicillin. The overnight cultures were used to inoculate 1L expression cultures (LB medium with the appropriate antibiotics: 25 μg/ml kanamycin and 200 μg/ml ampicillin) to an optical density of 0.2 at 600nm. The expression cultures were grown with shaking at 180–200 rpm at 28°C until an optical density of OD_600_ 0.5 was reached. By adding 1mM IPTG (Roth) (final concentration) recombinant protein expression was induced for 4h at 28°C with shaking at 180–200 rpm prior to harvest (5000 g, 20 min, 4°C). In a modified protocol, which in addition allowed the expression of 3kptC^C^, expression cultures were inoculated to an optical density of approximately 0.02 instead of 0.2, and 0.5mM instead of 1mM IPTG were added to induce protein expression. Harvested cell pellets were thawed on ice and resuspended in lysis buffer containing Pefabloc protease inhibitor (Roth) (1mM final conc.) to minimize protein degradation. Cell lysis was performed by sonication on ice (six 10-sec bursts with cooling pause between each burst). The lysate was then centrifuged at 10000g for 30min at 4°C to separate the soluble (supernatant) from the insoluble (pellet) proteins. Recombinant proteins from the supernatant, containing an N-terminal His_6_ tag, were purified using nickel-nitrilotriacetic acid (Ni-NTA; Qiagen) affinity chromatography under native (non-denaturing) conditions following the manufacturer´s instructions [45] The following buffers were used: Lysis buffer (50mM NaH_2_PO_4_ pH8.0; 300mM NaCl; 10mM imidazole), wash buffer (50mM NaH_2_PO_4_ pH8.0; 300mM NaCl; 20mM imidazole), elution buffer (50mM NaH_2_PO_4_ pH8.0; 300mM NaCl; 250mM imidazole). Polypropylene columns (5ml; Qiagen) packed with Ni-NTA Agarose (Qiagen) were used for purification. Purified proteins were dialyzed in 1xPBS buffer (136mM NaCl, 2.7mM KCl, 8mM Na_2_HPO_4_, 1.8mM KH_2_PO_4_, pH 7.4). The concentration of the recombinant proteins was determined by measuring the absorption at 280 nm using a NanoDrop Spectrophotometer ND-1000 (Peqlab) and taking into account the molar extinction coefficients of the proteins.

### Demonstration of *in vitro* isopeptide bond formation

Purified tag and catcher proteins were mixed each at the same molarity and incubated for the indicated time periods at 25°C with shaking at 500 rpm. Spontaneous isopeptide bond formation was monitored by performing time course experiments. 4xLaemmli buffer (200mM Tris-HCl pH6.8, 18% β-mercaptoethanol, 40% glycerol, 0.01% bromophenol blue, 8% SDS) was added to the samples prior to boiling at 95°C for 10 min. Proteins were separated on SDS-PAGE (12.5% Bis-Tris gel) and subsequently stained with Coomassie Brilliant Blue to visualize the formation of a covalent product resulting from the interaction between tag and catcher constructs over time. Besides visual inspection, quantitative analysis of the formed covalent product was performed by densitometry using the program ImageJ (https://imagej.nih.gov/ij/index.html). The mean values reflecting the band intensities were calculated in Microsoft-Excel and standard deviation values were added.

### Western blot

Besides Coomassie staining to detect the purified 6x-His tagged mCherry_only_ protein (pQE-9/mCherry expression construct), Anti-His peroxidase linked (Anti-His POD) Western Blot analysis was performed. After SDS-PAGE (12.5% Bis Tris gel) proteins were transferred to a nitrocellulose blotting membrane (GE Healthcare) using semi-dry electroblotting procedure. Immobilized proteins were detected after blocking the membrane with 5% milk powder solution in 1xTBS-T with Anti-His POD antibody (monoclonal anti-polyhistidine clone His-1 peroxidase conjugate; Sigma) in a 1:10000 dilution in 1% milk powder solution in 1xTBS-T. The peroxidase mediated oxidation of luminol allowed the specific detection of His-tagged proteins.

### CD-spectroscopy

Circular Dichroism (CD)-spectroscopy experiments to estimate the secondary structure of 4oq1^C^ were performed on a JASCO J-815 CD spectrophotometer equipped with a Peltier temperature control. The proteins were used at a concentration of 0.3 mg/ml, in 10mM sodium phosphate buffer pH 7. For CD data measurements 200μl protein was used in 1mm path length quartz cuvette. Wavelength scans between 185–260 nm were collected at 20°C using 1.0 nm band width, 0.1 nm step size, 20 nm/min scanning speed and a data integration time of 1 sec. Per measured sample six scans were acquired. The secondary structure analysis was done with the tool CDPro Analysis (part of Spectra Analysis software version 2.10.03, JASCO) using the algorithms CONTIN and SELCON3.

### Mass spectrometry

50μM of affinity-purified Catcher and 10μM of respective affinity-purified Tag-MBP in 1xPBS pH7.5 were incubated at 25°C, 500rpm for 24 hours before separating the proteins on SDS-PAGE (SDS gels: 10% separation gel and 5% stacking gel). Corresponding gel pieces for catcher-tag fusion products were in gel digested with trypsin (Thermo Fisher Scientific) in 50mM ammonium bicarbonate buffer pH 8 overnight. Resulting peptides were acidified to 0.1% formic acid and loaded on a nanoflow Ultimate 3000 HPLC (Dionex, Sunnyvale, CA, USA) for separation on EASY-Spray column (Thermo Fisher Scientific; C18 with 3 μm particle size, 15 cm x 75 μm) with a flow rate of 200 nl/min by increasing acetonitrile concentrations over 30 min. All samples were analyzed on an Orbitrap Fusion (Thermo Fisher Scientific) with the following settings: 2000 V spray voltage, 300–2000 (m/z) scan range, a maximum injection time of 50 ms, and an AGC target of 400.000 for first stage of mass analysis (MS^1^). The most intense ions were selected for collision induced dissociation with collision energy of 35%, a maximum injection time of 250 ms and an AGC target of 100 for second stage of mass analysis (MS^2^). Resulting spectra were analyzed with Proteome Discoverer 1.4 (Thermo Fisher Scientific). For database search either 4oq1^T^-MBP and 4oq1^C^ or 3kptC^T^-MBP and 3kptC^C^ sequences were combined with the *E*.*coli* uniprot database resulting in 4308 entries. For identification the thresholds were set on 1% FDR and allowed maximum two missed cleavages, oxidation of methionine was set as dynamic and carbamidomethylation as static modification for cysteines.

## Results and discussion

### System selection and structural stability of candidate systems

**Fig 3:**
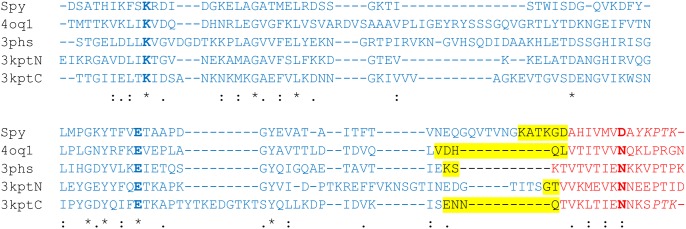
Structure-based multiple sequence alignment of the original Spy-system and the four constructs selected for the present study. The active site residues are shown in bold. Residues of the tag, catcher, and deleted regions are marked in red, blue, and yellow, respectively. A “*” marks a strictly conserved sequence position; “:” and “.” denote decreasing degrees of sequence similarity.

To assess the suitability of divergent CnaB-domains for the generation of tag/catcher systems, four domains were selected that exhibit less than 30% sequence identity to the original Spy-system [47] and to each other (Table 1). Fig 2 shows the structures of the parent CnaB-type domains and displays the tag and catcher regions as well as the residues deleted. Despite their low sequence identity, the structures display a similar fold, indicated by a backbone root mean square deviation (RMSD) of 1.8 to 2.2 Å relative to each other, but differ significantly in the length and structure of the loops connecting the conserved β-strands (Figs 2 and 3). One difference between the four candidate domains and the Spy-system is the isopeptide binding partner of the reactive Lys, which is Asp in the Spy-system, but Asn in the four candidate domains (Fig 3).

Molecular dynamics (MD) simulations were used to study the effect of splitting the parent domain into a tag/catcher system. The main objective here was to analyze the candidates’ structural stability *in silico* in order to exclude highly unstable split sites from further experimental analysis. This approach is inspired by our previous work on complexes between the SUMO-protein and small SIM peptides. For this system, 200 ns of MD simulation were sufficient to monitor dissociation of highly unstable binding modes [48].

**Fig 4:**
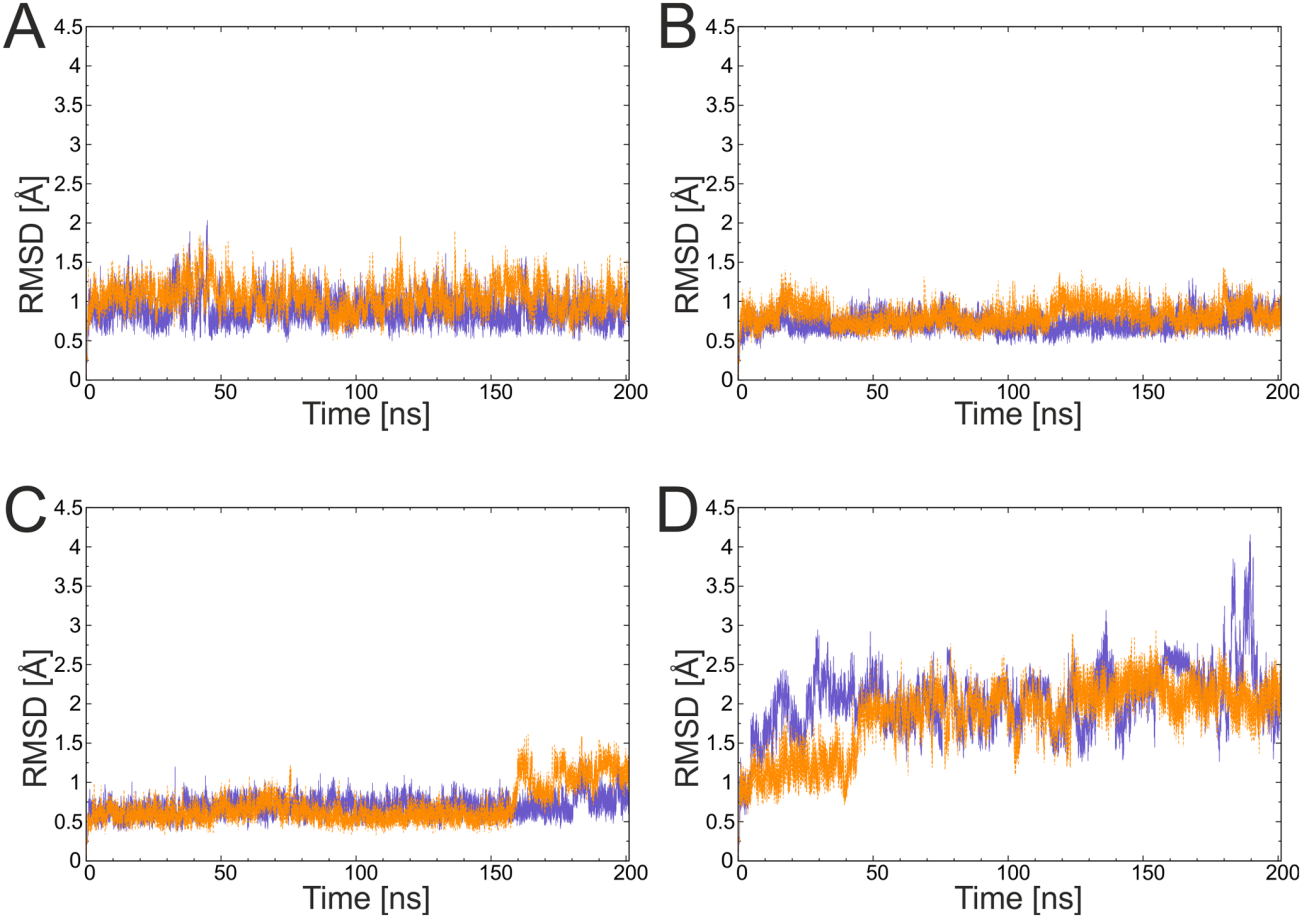
Conformational stability of the parent domains (violet) and tag/catcher (orange) constructs deduced from the molecular dynamics simulations. (A) 3phs, (B) 4oq1, (C) 3kptN, and (D) 3kptC. The RMSD was calculated for the backbone of the residues forming the core of the domains (see Methods for a definition of the core residues).

To investigate the overall stability of a system, the RMSD evolution over time is a common measure. Fig 4 shows the RMSD plots for the parent domains and tag/catcher complexes. Splitting the full-length domains into two chains did not significantly alter the overall flexibility as evident from similar RMSD values of the domain and tag/catcher constructs for all four systems investigated. Notably, 3kptC was intrinsically more flexible than the other three systems, which showed mean RMSD values of below 1.6 Å. None of the systems unfolded during the simulation and no dissociation of the tags was observed.

**Fig 5:**
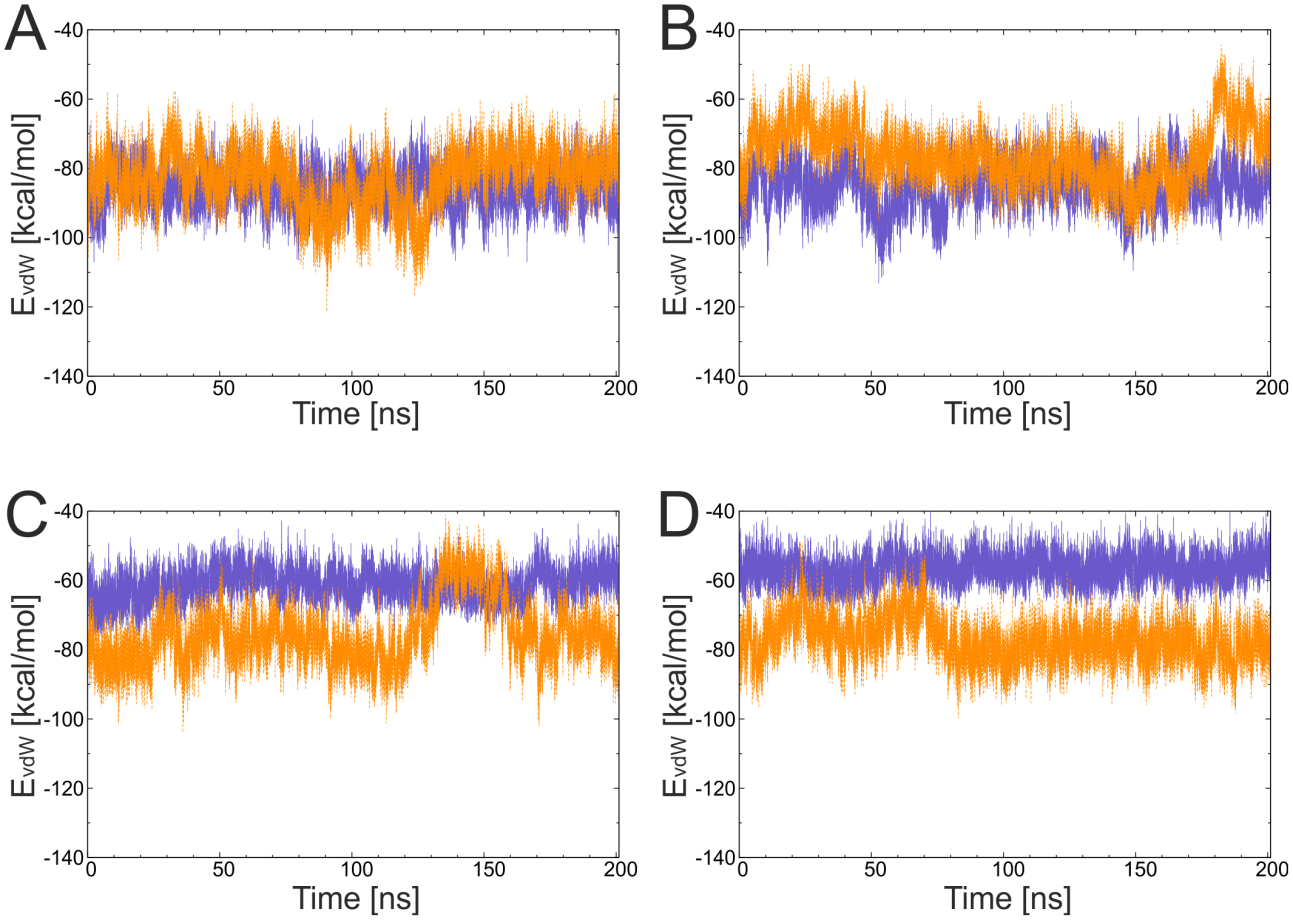
Evolution of van-der-Waals interaction energy of the tag in parent domains (violet) and tag/catcher (orange) constructs. (A) 3phs, (B) 4oq1, (C) 3kptN, and (D) 3kptC.

**Fig 6:**
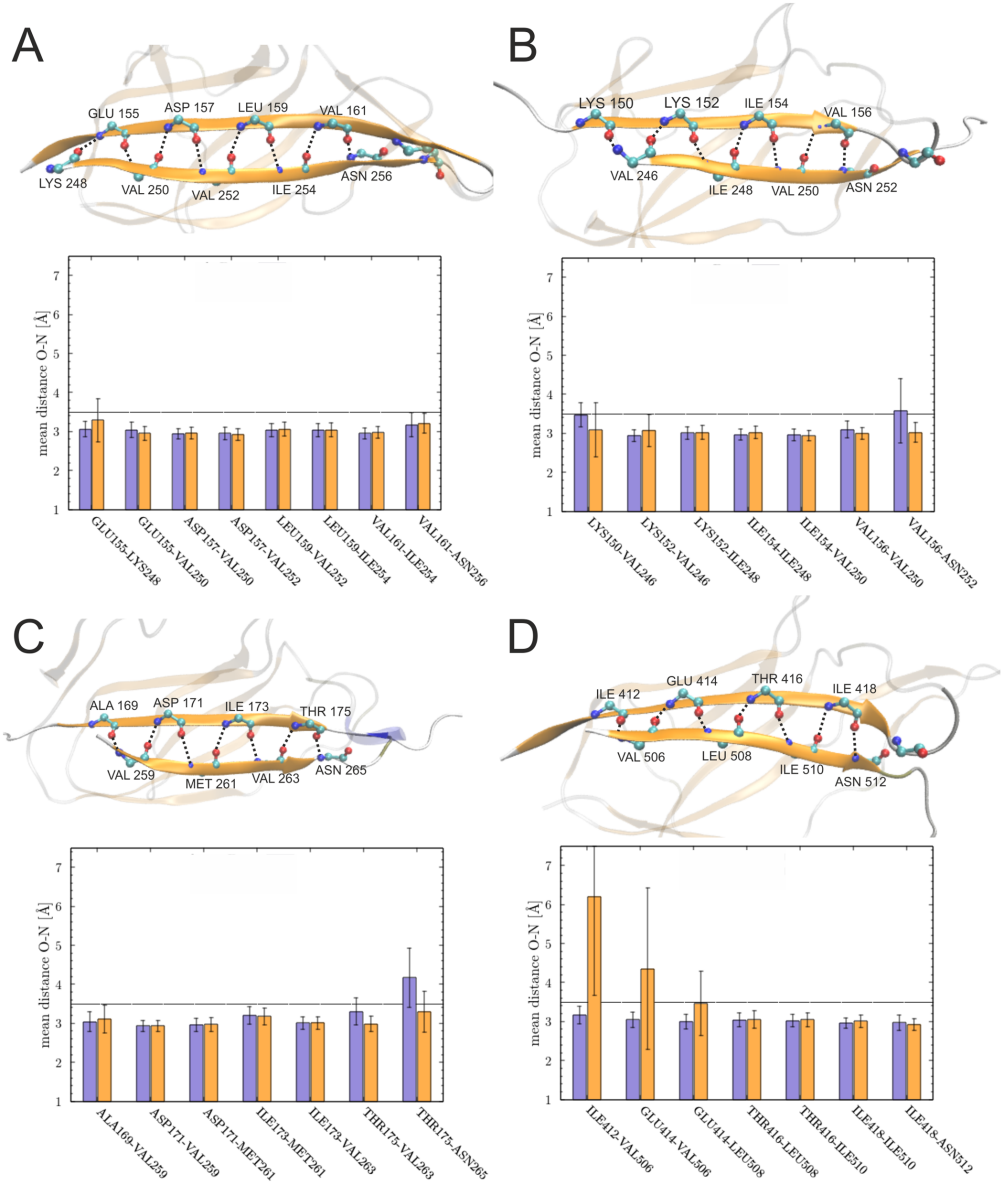
Hydrogen bonds between the tag and catcher region compared for domain (violet) and tag/catcher systems (orange). The structure presentations show the spatial arrangement of the hydrogen bond network and the diagrams show the mean distance observed over the simulation time between the participating residues for (A) 3phs, (B) 4oq1, (C) 3kptN, and (D) 3kptC. Residues located C-terminally of the reactive Asn position did not form β-sheet hydrogen bonds and are therefore not shown.

For a more detailed investigation the van-der-Waals interaction energy of the tag residues with the catcher moiety was calculated for the tag/catcher and original domain systems (Fig 5). Energy values for all systems were around -60 to -80 kcal/mol, and the tag/catcher systems exhibited similar or even more favorable interaction energies compared to the parent domains, as in the case of the two 3kpt-derived constructs. The interaction between tag and catcher was further analyzed by inspecting the backbone hydrogen bonds formed over the simulation time (Fig 6). Most hydrogen bonds stabilizing the parallel arrangement of the β-strands remained stable in all the systems (Fig 6) and splitting had only small effects on the stability of the hydrogen bonds. The only exception are the hydrogen bonds formed by the N-terminal tag residue V506 in 3kptC (Fig 6D), which became slightly more unstable upon splitting. However, in this system the loss of hydrogen bonds was compensated by the formation of new favorable van-der-Waals interactions (Fig 5D).

Taken together, the *in silico* analysis had the objective to examine the effect of splitting the protein domain into two parts. All four designed tag/catcher systems exhibited a similar overall dynamics compared to their parent domain and showed no large fluctuations of the newly emerging termini. This suggests that reasonable split points were selected and all four systems were subjected to further experimental investigation.

### Heterologous expression of recombinant proteins in *E*. *coli* M15 [pREP4] cells and purification via Ni-NTA affinity chromatography under native conditions

**Fig 7:**
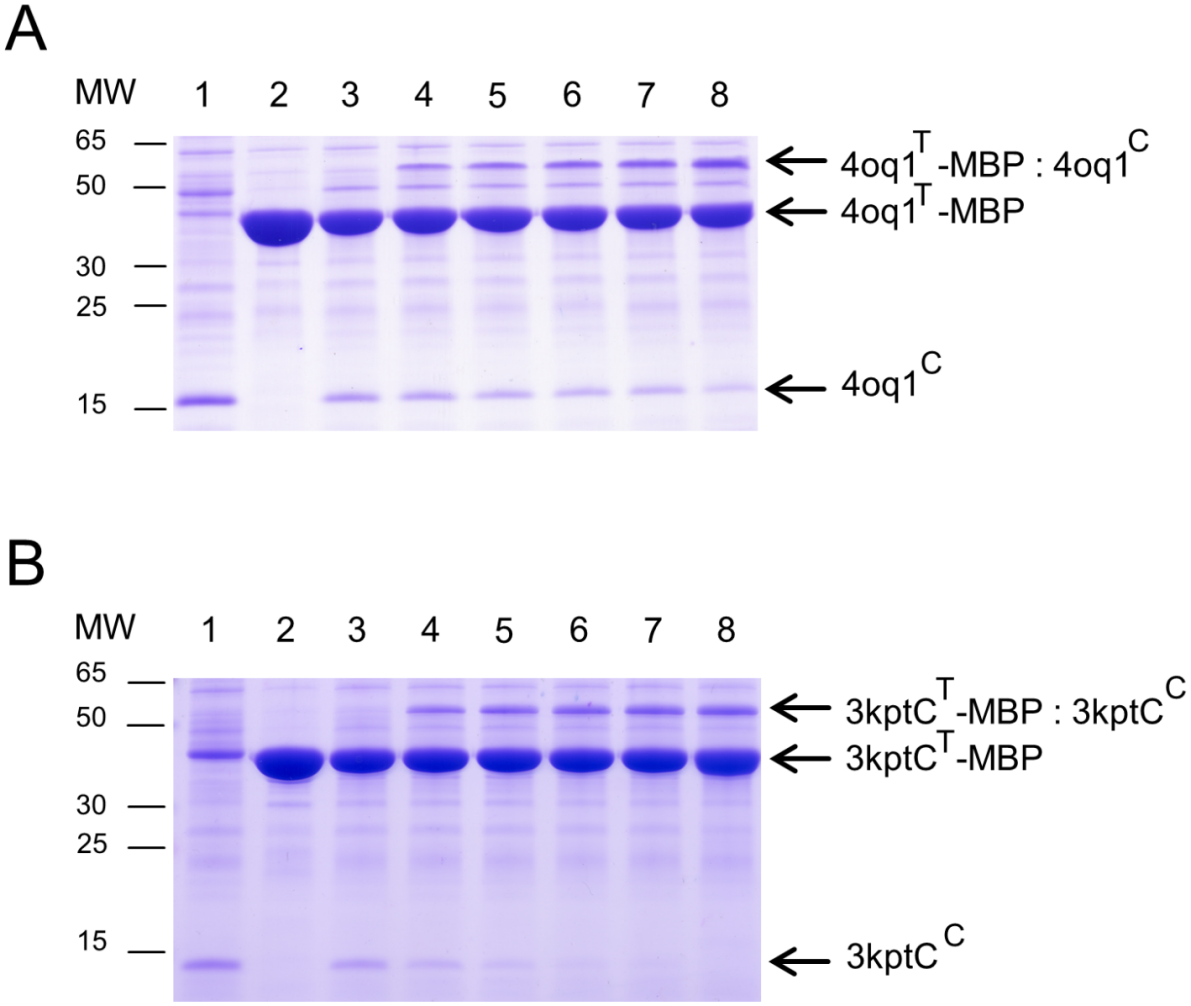
Covalent intermolecular bond formation assays for 4oq1^C^ / 4oq1^T^-MBP (A) and 3kptC^C^ / 3kptC^T^-MBP (B). (A) Purified 4oq1^T^-MBP and 4oq1^C^ were mixed each at 15 μM (final concentration) for 24h at 25°C with shaking at 500 rpm before boiling (10min, 95°C) and SDS-PAGE with Coomassie staining. 4oq1^T^-MBP and 4oq1^C^ reacted spontaneously to form a covalent product increasing over time (lane 1: catcher input (30μM), lane 2: tag input (30μM), lane 3: 0h, lane 4: 1h, lane 5: 2h, lane 6: 3h, lane 7: 4h, lane 8: 24h). Same volume of samples were loaded. MW stands for molecular weight (kDa). (B) Bond formation assay monitoring the reaction of 3kptC^T^-MBP and 3kptC^C^. All experimental settings as described in (A).

**Fig 8:**
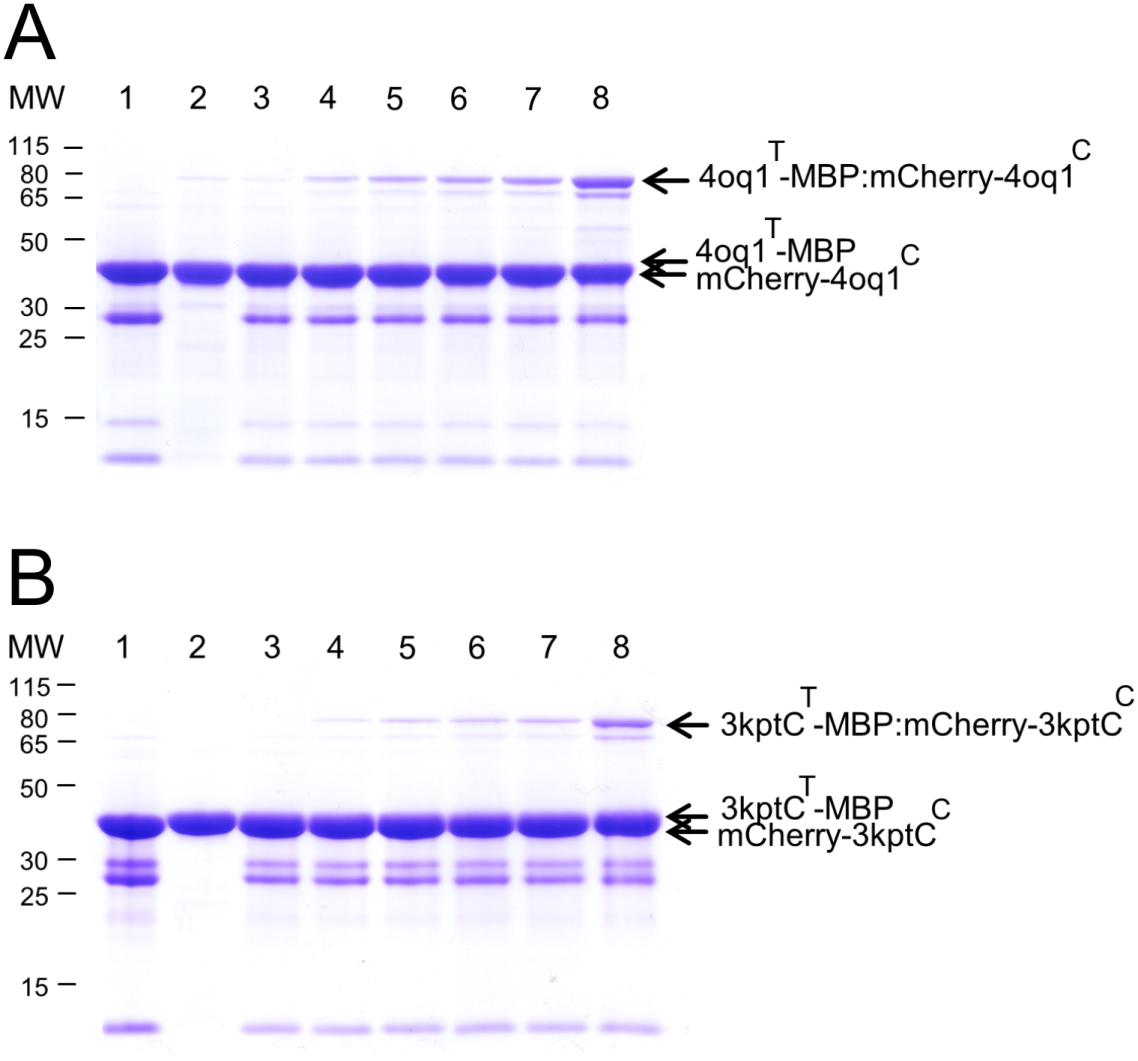
Covalent intermolecular bond formation assay between tag-MBP and respective mCherry-catcher proteins. Purified tag-MBP and mCherry-catcher proteins were mixed each at 15 μM (final concentration) for 24h at 25°C with shaking at 500 rpm before boiling (10min, 95°C) and SDS-PAGE with Coomassie staining. (A) Time course of 4oq1^T^-MBP and mCherry-4oq1^C^ reaction. (B) Time course of 3kptC^T^-MBP and mCherry-3kptC^C^ reaction. Spontaneous isopeptide bond formation was detected between the protein partners used. Covalent product increased over time. Lane 1: mCherry-catcher input (30μM), lane 2: tag input (30μM), lane 3: 0h, lane 4: 1h, lane 5: 2h, lane 6: 3h, lane 7: 4h, lane 8: 24h. Same volume of samples were loaded. MW stands for molecular weight (kDa).

The catcher constructs 3kptN^C^ and 3phs^C^ (approx. 13 kDa) showed no expression in the heterologous host *E*. *coli* M15 [pREP4] under tested conditions (S1 Fig). Only 4oq1^C^ and 3kptC^C^ (approx. 13kDa) could be detected in the Coomassie stained gel, indicating a successful expression in M15 [pREP4] *E*. *coli* cells (S1 Fig). All tag-MBP constructs (3kptN^T^-MBP, 3kptC^T^-MBP, 3phs^T^-MBP, 4oq1^T^-MBP; approx. 44 kDa), however, were well expressed as soluble proteins (S1 Fig). To test whether the properties of the short catcher constructs can be enhanced by the presence of a fusion partner, mCherry-catcher fusion proteins were cloned. These fusion constructs more closely reflect the situation of future applications, in which the catchers will be fused to the C-terminus of another protein to mediate its covalent linkage to a second protein containing the tag. In the present setup such a fusion construct was created by attaching mCherry fluorescent protein to the catcher. The mCherry-catcher fusion proteins (approx. 40 kDa) were expressed efficiently as soluble proteins in M15 [pREP4] *E*. *coli* cells (S2 Fig). All expressed proteins were successfully purified by Ni-NTA affinity chromatography under native conditions (see input samples in Figs 7 and 8 and S3 Fig). We noted additional bands in the gels, which in part represent impurities due to the one-step purification procedure. However, some bands also reflect degradation products predominantly coming from degradation of the mCherry fusion partner (Fig 8 and S3 Fig). We have investigated the isolated mCherry in more detail to verify that this protein exhibits an intrinsic instability under the present experimental conditions (S4 Fig). With these purified tag-MBP and mCherry-catcher fusion proteins covalent isopeptide bond formation assays were performed to test for intermolecular protein-protein linkage.

### *In-vitro* isopeptide bond formation assay

To test whether the split-protein tag/catcher domains are able to form an intermolecular isopeptide bond when the two parts recognize each other and reconstitute, *in vitro* experiments were performed. Isopeptide bond formation for the 4oq1- and 3kptC-system was tested using the isolated catchers (4oq1^C^, 3kptC^C^) as well as the mCherry-catcher fusion (mCherry-4oq1^C^, mCherry-3kpt^C^). Results showed that both split-protein systems were functional and the interaction between catcher and tag resulted in a covalent product that was resistant to boiling (10 min at 95°C) under reducing conditions and with SDS thus suggesting a covalent association (Figs 7 and 8). Using the mCherry-catcher instead of the short catcher construct did not significantly affect the coupling efficiency of the 4oq1- and 3kptC-system (Figs 7 and 8).

To verify that both educts—catcher and tag—are actually present in the new band that emerges over time in the gels, we have performed mass spectrometric analyses for the 4oq1- and 3kptC-systems (S5 Fig). In the case of the 4oq1 product band analysis, 4oq1^T^-MBP was detected with 84.22% amino acid coverage whereas 4oq1^C^ had a coverage of 74.77% (S5 Fig, S7 Table). The analysis of the 3kptC product band revealed a 93.53% coverage of the 3kptC^T^-MBP and 88.46% coverage for the corresponding 3kpt^C^ (S5 Fig, S8 Table). Taking together, for both systems– 4oq1 and 3kptC—the reaction partners, catcher and tag, were identified in the respective product bands with high confidence, underlining the assumption that the new arising SDS-PAGE band is indeed the product of both catcher and tag systems.

The presence of a covalent linkage was inferred from the stability of all products to boiling in SDS (95°C, 10 min) prior to running the gels. This procedure was done with all samples and is a widely used test to assess a covalent linkage of the educts. This approach has also been used to verify covalent linkage in the rated tag/catcher systems Spy [19], Snoop [33], and Sdy [35].

As a further test to verify the presence of a covalent linkage, we have now also repeated the assays for mutated tag or catcher proteins lacking the active site residues directly involved in isopeptide bond formation according to the respective CnaB crystal structures [17, 49] (N252A, K155A, E222Q for 4oq1 and N512A, K417A, E472Q for 3kptC). No product band is observed in these systems suggesting an essential role of the isopeptide bond for the formation of stable complexes (S6 and S7 Figs).

### Lack of reactivity for 3kptN and 3phs split protein systems

The two remaining catchers 3kptN and 3phs, however, were inactive as mCherry fusion proteins as evident from the absence of new product band in the gel (S3 Fig). The fact that isolated catchers 3kptN^C^ and 3phs^C^ cannot be expressed in a soluble form and that they are non-reactive as part of mCherry fusion proteins most likely indicates that these two constructs are not proper folded. 3kptN and 3kptC were derived from the CNA2 and CNA3 domains of the *Bacillus cereus* major backbone pilin BcpA (Table 1). The difference in reactivity observed between 3kptN and 3kptC suggests that not all domains of backbone pilins are equally well suited for the design of tag/catcher systems. This is in line with the previous observation that the N-terminal domain of some backbone pilins is labile and easily lost by proteolysis [50]. In addition, the N-terminal domains of *B*. *cereus* BcpA and *S*. *pneumoniae* RrgB form intra-domain isopeptide bonds during pilus assembly only, but not in the recombinant proteins [49, 51, 52]. The present study suggests that not only the CNA1 domain of BcpA, but also the CNA2 domain, which served as template for the design of 3kptN, exhibits only limited stability as isolated domain or at least when split into a tag/catcher system. The stability of 3kptN^C^ and 3phs^C^ might be improved in future by a variation of construct length. However, we did not perform a systematic optimization of these constructs because the major aim of our investigation was to establish a versatile screening protocol that allows screening a large number of CnaB-domains as candidates for the design of tag/catcher systems.

### Properties of the functional 4oq1 and 3kptC split-protein systems

In comparison with the extensively optimized Spy- and Snoop-system, the 4oq1 and 3kptC constructs investigated in this study are inefficient and slow with regard to isopeptide bond formation between the split protein partners. Whereas the covalent reaction of tag and catcher occurs efficiently in high yield within minutes in the established systems [19, 33], the covalent product seen after 1h of interaction between 4oq1^C^ and 4oq1^T^ and between 3kptC^C^ and 3kptC^T^ was weak but increasing over time (Figs 7 and 8). The spontaneous intermolecular covalent reaction between 4oq1^T^ and mCherry-4oq1^C^ started within 5 to 10 minutes but only to a small extent (S8B Fig panel I) and the catcher and tag molecules used as input were not fully consumed even after 4 hours and 24 hours reaction time, respectively (Fig 7). The same is true for the 3kptC-system where catcher and tag indeed started forming a covalent complex within minutes but only to a very small extent (S9B Fig panel I). Covalent product formation increased continually over time (Fig 8B, S9B Fig panel I) but did not reach reconstitution levels known from the Spy- and Snoop-system.

Slow isopeptide bond formation has also been described for the SpaD protein from *Corynebacterium diphtheriae*, in which full formation of the three isopeptide bonds required 24 h at 37°C or 72 h at room temperature [52]. Thus, the slow isopeptide bond formation observed for 4oq1 and 3kptC might be an intrinsic property of these domains. This aspect might be investigated in future in more detail by testing the rate of isopeptide bond formation directly in the parent CnaB domains.

However, the low reactivity might also result from the splitting into tag and catcher. In this context, the low reconstitution levels observed might indicate a folding problem of the catcher constructs, i.e. a portion of the catcher molecules might be misfolded and therefore unable to interact with the tag. To address this point in more detail, we have measured a CD-spectrum of 4oq1^C^. We have used the construct without mCherry fusion part to avoid that the signal in the CD-spectrum becomes dominated by the larger fusion domain. The spectrum exhibits a broad minimum from 205 to 225 nm indicative of a folded protein (S10 Fig). Estimation of the β-sheet content using two different algorithms resulted in values of 38–39%. Based on an inspection of the 4oq1 crystal structure one would expect a β-sheet content of 43% for the 4oq1^C^ moiety. Thus, the β-sheet content observed in the CD-spectrum is roughly in the range that one would expect for a folded 4oq1^C^ and there is no evidence for the presence of large amounts of unfolded protein, which would explain the low reconstitution levels observed.

In addition, we have also tested whether modified 4oq1^C^ constructs, which were either C-terminally extended by four residues (V241-Q244) or truncated by two residues (Q239-L240), allow for an enhanced reconstitution. The extended catcher performed similarly to the original construct, whereas the truncated catcher performed worse (data not shown).

An alternative source for poor reactivity might be an inappropriate length of the tags. We have addressed this potential source for reduced product yield in more detail as described below.

### Influence of the tag properties on isopeptide bond formation

**Fig 9:**
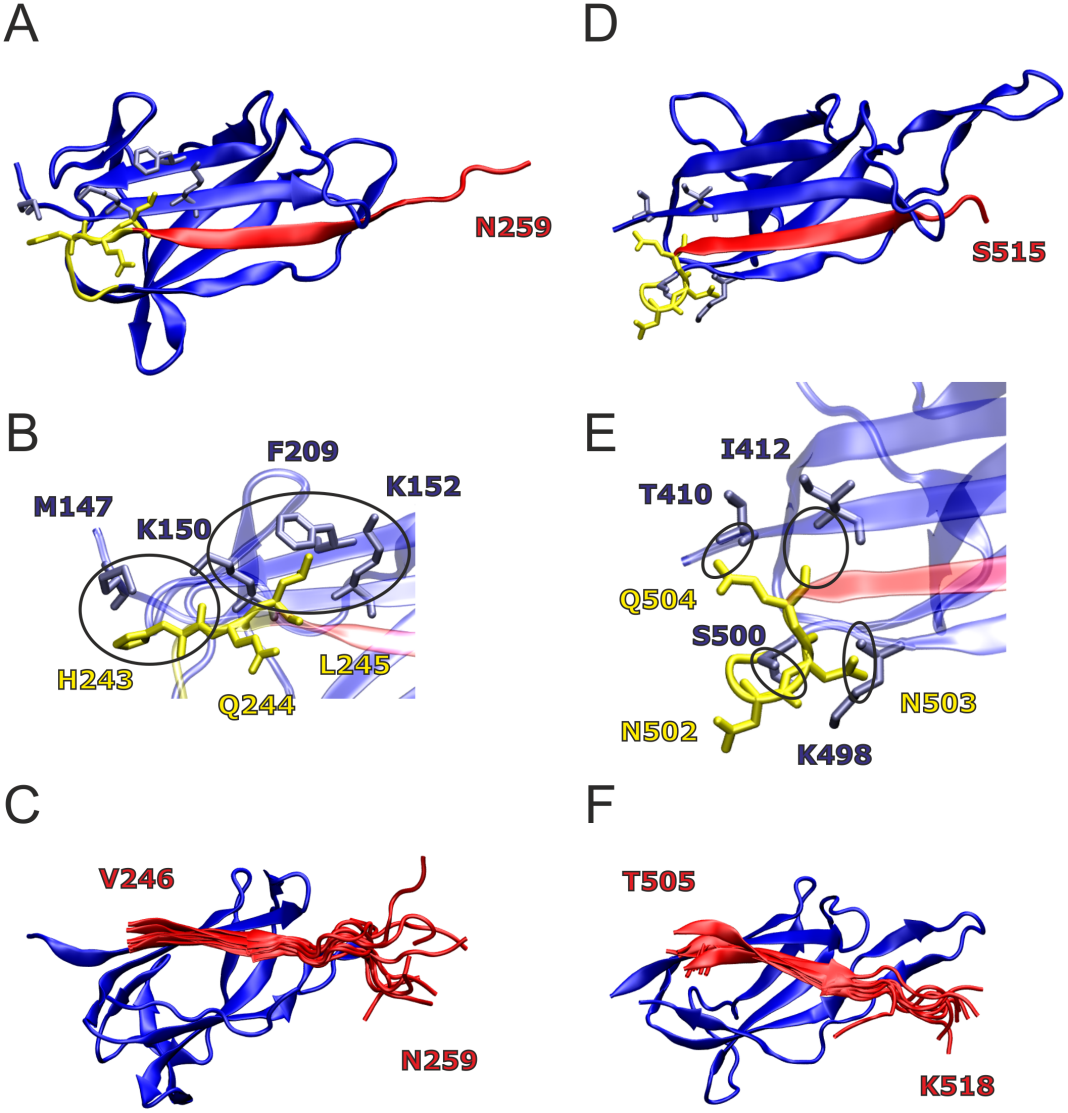
Candidate sites for modification of 4oq1^T^ and 3kptC^T^ (catcher region: blue; tag region: red; linking region: yellow). (**A**) 4oq1 crystal structure with residues H243-L245 of the linker sequence shown in yellow sticks, interacting residues in the catcher region within 4 Å are shown in blue sticks. (**B**) Enlargement showing the interacting residues in detail. Circles denote specific interactions. (**C**) Overlay of 11 tag peptide structures from the 4oq1 MD simulation; for the sake of clarity, only one catcher structure is shown. (**D**) 3kptC crystal structure with residues N502-Q504 of the linker sequence shown in yellow sticks, interacting residues in the catcher region within 4 Å are shown in blue sticks. (**E**) Enlargement showing the interacting residues in detail. Circles denote specific interactions. (**F**) Overlay of 11 tag peptide structures from the 3kptC MD simulation; for the sake of clarity, only one catcher structure is shown.

Variants of the 4oq1^T^ and 3kptC^T^ sequences were derived from analysis of the respective crystal structure and from dynamic properties observed during the MD simulation (Fig 9). An inspection of the 4oq1 crystal structure indicated that the tag peptide might gain additional interactions with the catcher by an N-terminal extension of the tag sequence that started at V246 in the original construct (Fig 9A): An inclusion of L245 would extend the parallel β-sheet formed between tag and catcher. In addition, L245 shows favorable hydrophobic interactions with neighboring catcher residues; its sidechain protrudes between the aliphatic parts of the K150 and K152 side chains of the adjacent strand and contacts F209 of the second next strand (Fig 9B). A longer N-terminal extension by three residues (H243-L245) would additionally allow H243 to contact M147 and to form polar interactions between its backbone carbonyl group and the charged ammonium group of K150. In contrast, Q244 is oriented towards the solvent in this structure and thus provides no further direct stabilization. Thus, from the analysis of the static structure two N-terminal extensions of the tag peptide comprising either L245 or H243-L245 were suggested (4oq1^T^(L) and 4oq1^T^(HQL)).

In contrary to the N-terminal extension, a C-terminal extension of 4oq1^T^ did not appear reasonable, because even the residues present in the original construct formed little or no interactions with the catcher. This observation is further substantiated by the results of the MD simulation (Fig 9C) showing that the C-terminal residues of the tag are highly flexible.

Such flexible regions may hamper the formation of a binding-competent conformation or lead to nonspecific interactions between tag and catcher. To address whether the C-terminal residues are dispensable for binding or do even negatively affect the interaction as observed for SdyTag [35], a C-terminal truncation of the tag by three residues (R257-N259) was proposed (4oq1^T^(ΔRGN)).

In 3kptC, residues E501-Q504 were removed in the splitting procedure (Figs 2 and 9D). A closer inspection showed that residue Q504 and N503 form tight interactions with residues of the catcher moiety, whereas N502 does not form specific interactions (Fig 9E). This prompted us to extend 3kptC^T^ by N503 and Q504 (3kptC^T^(NQ)) to allow for additional interactions with the catcher.

The C-terminal residues in the 3kptC crystal structure is S515 (Fig 9D). The native protein comprises 552 residues and its hydrophobic C-terminus contains an LPXTG-motif involved in cell wall anchoring [23]. To avoid potential aggregation problems, we did not consider the respective region in our original 3kptC^T^ construct, but instead added three artificial residues (P516-K518) derived from the SpyTag, where they enhanced reactivity [19]. Inspection of the dynamics of 3kptC^T^ revealed that residues P516-K518 were less flexible compared to the C-terminal residues of 4oq1^T^ (Fig 9C and 9F) and formed interactions with 3kptC^C^. Therefore, we tested, whether the tag-catcher interaction can be further enhanced by replacing the terminal “PTK” sequence stretch by the hydrophobic “GWI” sequence (3kptC^T^(GWI)) corresponding to residues 516–518 of the native BcpA protein.

**Fig 10:**
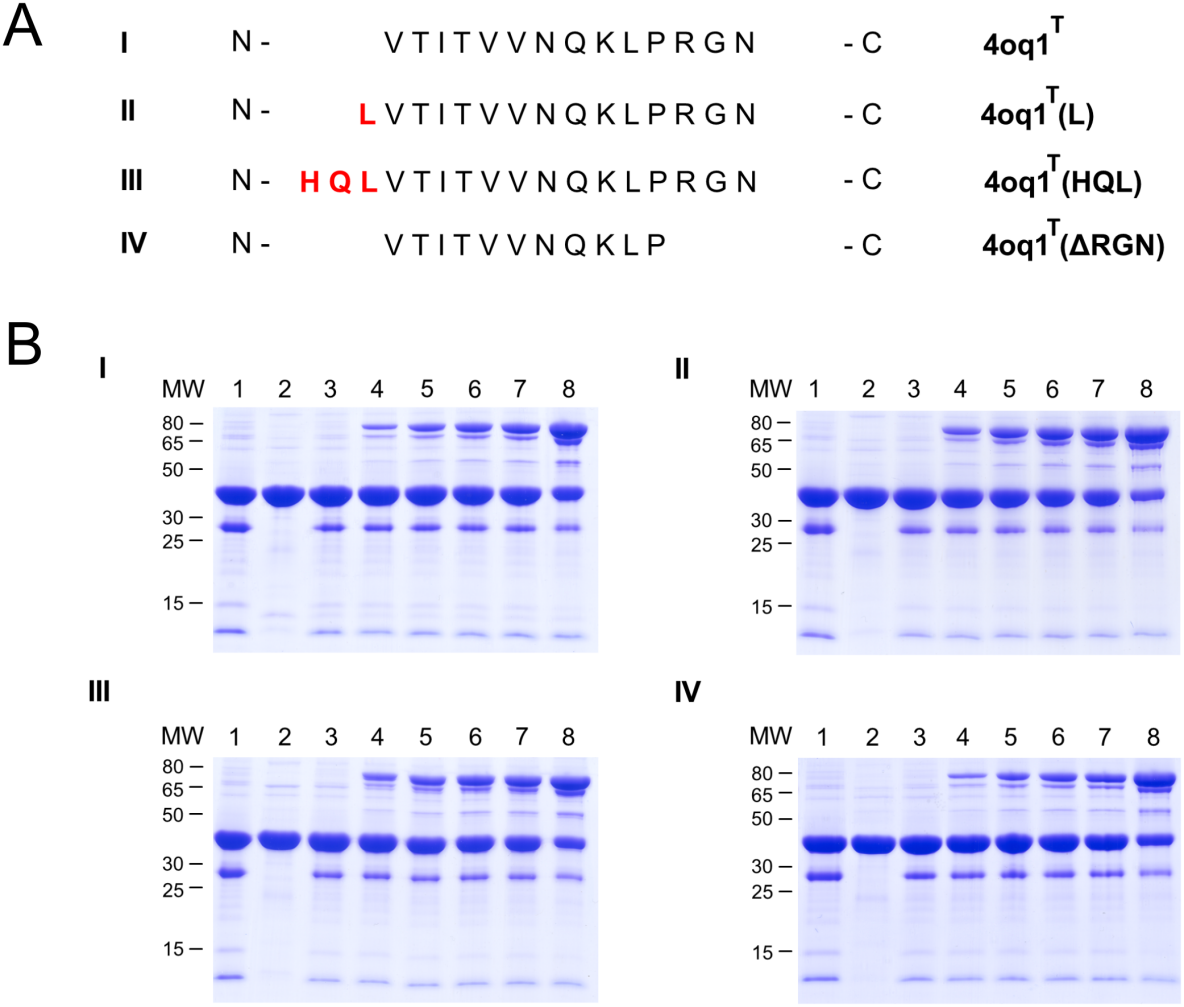
Comparative analysis of different 4oq1^T^-MBP variants. (A) Sequence alignment of different 4oq1^T^-MBP variants (I: 4oq1^T^ wildtype (residues V246-N259), II: N-terminal L245 extension of 4oq1^T^, III: N-terminal H243-Q244-L245 extension of 4oq1^T^, IV: C-terminal R257-G258-N259 truncation of 4oq1^T^). (B) Comparative covalent intermolecular bond formation assay between different 4oq1^T^-MBP variants and mCherry-4oq1^C^ (0h-24h). Purified 4oq1^T^ variants and mCherry-4oq1^C^ proteins were mixed each at 15 μM (final conc.) for 24h at 25°C with shaking at 500 rpm before boiling (10min, 95°C) and SDS-PAGE with Coomassie staining. Interaction I: mCherry-4oq1^C^ + 4oq1^T^ (wildtype), interaction II: mCherry-4oq1^C^ + 4oq1^T^ (L), interaction III: mCherry-4oq1^C^ + 4oq1^T^ (HQL), interaction IV: mCherry-4oq1^C^ + 4oq1^T^ (ΔRGN). (lane 1: mCherry-catcher input (30μM), lane 2: tag input (30μM), lane 3: 0h, lane 4: 1h, lane 5: 2h, lane 6: 3h, lane 7: 4h, lane 8: 24h). Same volume of samples were loaded. MW stands for molecular weight (kDa).

**Fig 11:**
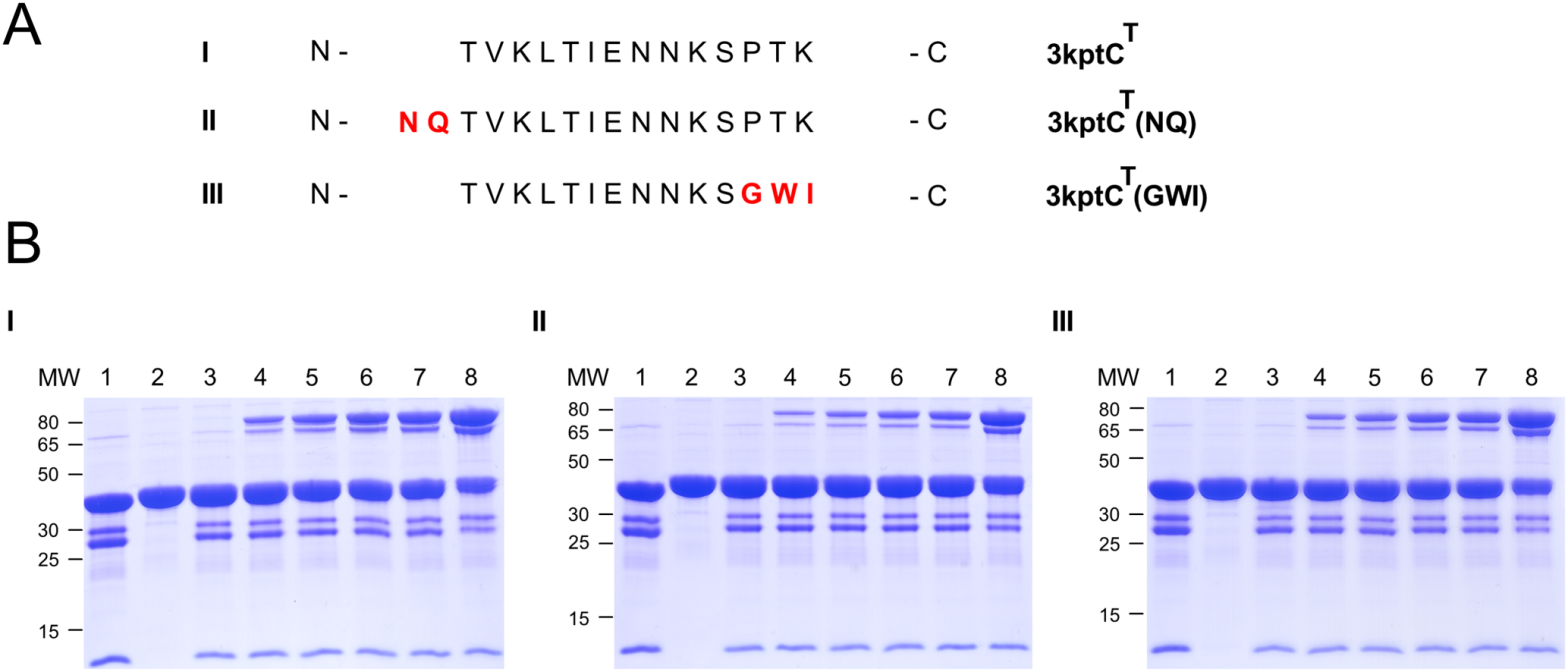
Comparative analysis of different 3kptC^T^-MBP variants. (A) Sequence alignment of different 3kptC^T^-MBP variants (I: 3kptC^T^ wildtype (residues T505-K518), II: N-terminal N506-Q507 extension of 3kptC^T^, III: C-terminal GWI instead of PTK in 3kptC^T^). (B) Comparative covalent intermolecular bond formation assay between different 3kptC^T^-MBP variants and mCherry-3kptC^C^. Purified 3kptC^T^ variants and mCherry-3kptC^C^ proteins were mixed each at 15 μM (final conc.) for 24h at 25°C with shaking at 500 rpm before boiling (10min, 95°C) and SDS-PAGE with Coomassie staining. Interaction I: mCherry-3kptC^C^ + 3kptC^T^ (wildtype), interaction II: mCherry-3kptC^C^ + 3kptC^T^ (NQ), interaction III: mCherry-3kptC^C^ + 3kptC^T^ (GWI). (lane 1: mCherry-catcher input (30μM), lane 2: tag input (30μM), lane 3: 0h, lane 4: 1h, lane 5: 2h, lane 6: 3h, lane 7: 4h, lane 8: 24h). Same volume of samples were loaded. MW stands for molecular weight (kDa).

**Fig 12:**
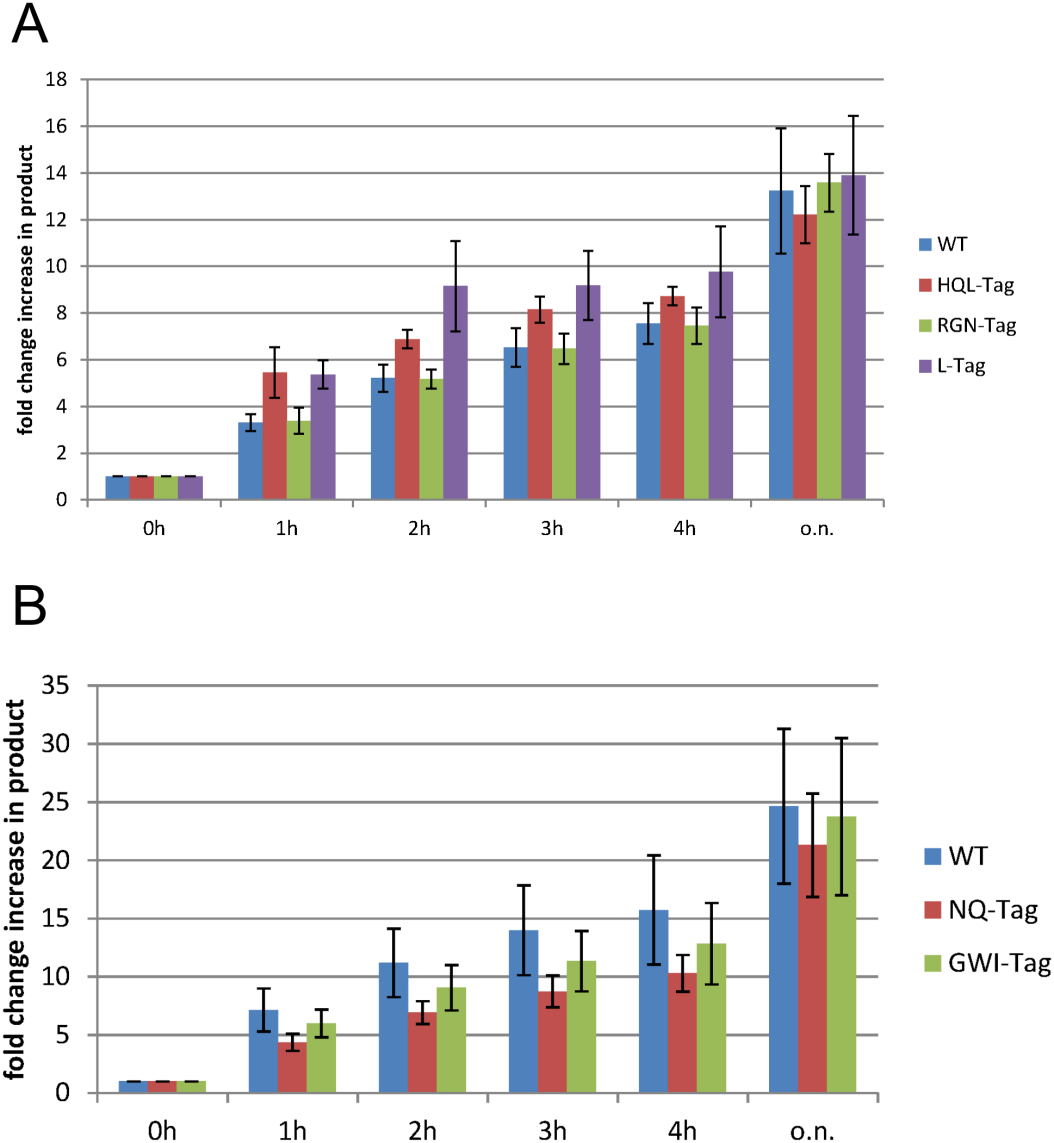
Bar diagrams summarizing the quantified reconstitution rates for (A) 4oq1 variants and (B) 3kptC variants. For each time point, the reconstitution of the wiltype-tag and different tag-variants is compared (please refer to Fig 11 for the exact sequence of the different tag variants). Each value represents the average over 3–4 independent experiments. o.n. = over night (24 h). Purified mCherry-catcher (15μM final conc.) and respective purified tag-MBP (15μM final conc.) were mixed at 25°C with shaking (500rpm) and a time course of covalent bond formation was performed (0h, 1h, 2h, 3h, 4h, o.n. = over night). Samples were boiled (10min, 95°C) prior to gel loading. Four independent assays per catcher-tag pair were performed (triplicate for mCherry-3kptC^C^-3kptC^T^(NQ)) and analyzed via densitometry (ImageJ). Based on the band intensities in the gel mean values and standard deviation were calculated.

Based on the structural analysis, the respective modifications at the N- and C- terminus of 4oq1^T^ and 3kptC^T^ were generated. After cloning, recombinant expression and purification of the various tag variants, comparative covalent bond formation assays were performed. All experiments were repeated 3–4 times and the intensity of the product band on the gel was quantified using densitometry. Representative gels showing the rate of isopeptide bond formation on two different time scales are presented in Figs 10 and 11 as well as in S8 and S9 Figs, and results are summarized in Fig 12. The results indicate that neither the C-terminal shortening of 4oq1^T^ (4oq1^T^(ΔRGN)) nor the N-terminal extensions (4oq1^T^(L) and 4oq1^T^(HQL)) have a significant effect on the overall amount of product formed after 24h (Fig 12). However, The N-terminal extensions (4oq1^T^(L) and 4oq1^T^(HQL)) displayed slightly improved rate for isopeptide bond formation, as evidenced from the larger amount of product formed within 1–4 hours (Fig 12).

Consequently this is a good starting point for further optimization. In summary, the experiments above suggest that varying the length of the tag by only several amino acids has only a small effect on the overall reactivity of the 4oq1-system while the reaction rate was slightly improved.

For the 3kptC-system a replacement of the C-terminal “PTK” sequence in 3kptC^T^ by “GWI” did not significantly alter the reaction rate and a N-terminal extension of the 3kptC^T^ sequence by two residues (“NQ”) even resulted in a slightly decreased reactivity compared to the wildtype 3kptC-system (Figs 11 and 12, S9 Fig). We compared these results obtained for the 3kptC-system (Fig 11) to the effects detected for 4oq1^T^ (Fig 10) and SpyTag [19] modifications.

The importance of the C-terminal residues for the SpyTag's reactivity has been demonstrated clearly: The original tag (sequence: “AHIVMVDA”) showed only 28% reconstitution after 30 minutes [19]. A C-terminal extension by three residues resulted in significantly enhanced reconstitution and the degree of improvement was sequence dependent (“AHIVMVDA**YKP**” performed better than “AHIVMVDA**GSR**”) [19]. Further optimization by adding two more residues (“AHIVMVDAYKP**TK**”) resulted in the final SpyTag with >80% reconstitution under the same conditions [19].

In case of 3kptC^T^, the constructs carrying either “PTK” or “GWI” as C-terminal residues exhibited a similar reactivity (Fig 11). This might either indicate that the tag-catcher interaction of this peptide stretch is rather independent of sequence or that these residues are dispensable for the interaction at all. The latter situation was observed for the 4oq1^T^ construct, which tolerated deletion of the three C-terminal residues without losing reactivity (Fig 10). This finding for 4oq1^T^ is remarkable, because it suggests that the respective residues might be replaced by a designed peptide sequence to enhance tag-catcher affinity in future.

With respect to an N-terminal tag extension, 4oq1^T^ and 3kptC^T^ showed different trends: An extension by “L” or “HQL” enhances reactivity of 4oq1^T^, whereas a slightly decreased reactivity is observed for 3kptC^T^ extended by the “NQ” sequence. A similar observation such as for 3kptC^T^ has also been made for the SpyTag, for which an N-terminal extension by the “GD” sequence slightly reduced reactivity [19]. Our results together with previous work from others [18, 19] suggest that the N-terminal length of the tag peptide has only a limited effect on the reactivity of the systems.

### Conclusions and outlook

The aim of our study was to establish a versatile screening protocol that allows assessing CnaB-domains as candidates for the design of tag/catcher systems. We have exemplarily selected four CnaB domains of low sequence similarity and characterized the resulting tag/catcher systems by computational and experimental methods.

As first step of our strategy we performed comparative computer simulations of the intact CnaB-domains and of constructs split into tag and catcher. This setup aimed to identify those systems, which become instable upon splitting into tag and catcher. This setup is based on the assumption that the catchers are folded in a binding-competent conformation. However, subsequent experiments revealed that the instability of the catcher represents a major problem for at least two of the systems investigated (3phs, 3kptN). Thus, additional computational investigations addressing the conformational stability of the isolated catchers should supplement the present approach in future.

The second step of our approach was to establish an experimental screening procedure, which aimed to identify those systems that remain functional after splitting. Most tests were performed for tags and catchers as parts of fusion proteins. These fusion constructs more closely reflect the situation in future applications, in which the catchers will be fused to the C-terminus of another protein to mediate its covalent linkage to a second protein containing the tag. However, mCherry turned out to be problematic as a fusion partner due to its high degradation tendency under the experimental conditions of the present study. Thus, mCherry should be replaced by another fusion protein (e.g. SUMO [30]) in future screening procedures. This would also allow resolving the problem that both reactants (tag-MBP and mCherry-catcher) have almost the same size hampering a quantification of the reactant bands in the gels.

Our screening approach revealed that two of the systems remained functional after splitting (4oq1, 3kptC), whereas no activity was detected for the other two systems (3kptN, 3phs). The fact that isolated catchers 3kptN^C^ and 3phs^C^ cannot be expressed in a soluble form and that they are non-reactive as part of mCherry fusion proteins most likely indicates that these two constructs are not folded properly. Their stability might be improved in future by testing different lengths of these constructs. Therefore, the number of two functional systems out of four systems tested here marks a lower limit of functional systems that can be obtained by this screening approach.

For the two functional systems we also performed some initial test to assess the role of construct length on the reactivity of the systems. This was done for the length of 4oq1^C^, 4oq1^T^, and 3kpt^T^. Our study suggests that the systems are rather robust with respect to the exact splitting site and the reactivity of the different constructs is rather similar. This observation is in line with the findings of other groups that and optimization of tag-catcher-systems is not trivial and requires a subsequent optimization of reactivity will be required for most split-protein systems. In addition to the variation of tag and catcher length, further construct optimization may also include removal of surface exposed hydrophobic amino acids [19], stabilization of secondary structure [33], or optimization of electrostatic interactions [35]. Previous studies also indicate that a variation of the solvent conditions (buffer, pH, temperature) can significantly affect reaction rates [19].

In summary, the present study reports a screening strategy for the detection of functional tag-catcher systems. Our findings indicate that splitting into tag and a catcher moiety is tolerated by a significant portion of the naturally occurring CnaB-domains, thus providing a large reservoir for the design of novel tag/catcher systems.

## Supporting information

S1 Fig Time course of heterologous expression of catcher and tag proteins in M15 [pREP4] *E*.*coli* cells. (PDF)

S2 Fig Time course of heterologous expression of mCherry-catcher fusion proteins in M15 [pREP4] *E*.*coli* cells. (PDF)

S3 Fig Covalent intermolecular bond formation assay between tag-MBP and respective mCherry-catcher proteins. (PDF)

S4 Fig Purification of recombinantly expressed mCherry_only_ protein via Ni-NTA affinity chromatography. (PDF)

S5 Fig Mass spectrometric analysis of 4oq1 and 3kpt product band. (PDF)

S6 Fig Covalent bond formation assays of 4oq1 active site mutants. (PDF)

S7 Fig Covalent bond formation assays of 3kptC active site mutants. (PDF)

S8 Fig Rates of isopeptide bond formation of 4oq1^T^-MBP variants on the minute scale. (PDF)

S9 Fig Rates of isopeptide bond formation of 3kptC^T^-MBP variants on the minute scale. (PDF)

S10 Fig Far-UV CD-spectrum of 4oq1^C^. (PDF)

S1 Table Primers used in the present study. (PDF)

S2 Table Cloning scheme for mCherry-GSGSGSG-Catcher constructs. (PDF)

S3 Table Cloning scheme for 4oq1T-MBP variants (GSGESG linker and MBP sequence from pMAL-c2 vector). (PDF)

S4 Table Cloning scheme for 3kptCT-MBP variants (GSGESG linker and MBP sequence from pMAL-c2 vector), (PDF)

S5 Table Cloning scheme for non-reactive 4oq1 variants (GSGESG linker and MBP sequence from pMAL-c2 vector). (PDF)

S6 Table Cloning scheme for non-reactive 3kptC variants (GSGESG linker and MBP sequence from pMAL-c2 vector). (PDF)

S7 Table Peptides identified from mass spectrometric analysis after tryptic digestion of the 4oq1 product band. (XLSX)

S8 Table Peptides identified from mass spectrometric analysis after tryptic digestion of the 3kptC product band. (XLSX)

S1 Text Sequences. (PDF)
