# Supplementary material for: Probing the potential of CnaB-type domains for the design of tag/catcher systems
Source: PLoS One. 2017 Jun 27;12(6):e0179740. doi: 10.1371/journal.pone.0179740 (PMC5487036; doi:10.1371/journal.pone.0179740)
Supplement: S1 Fig — (PDF) [file pone.0179740.s001.pdf]

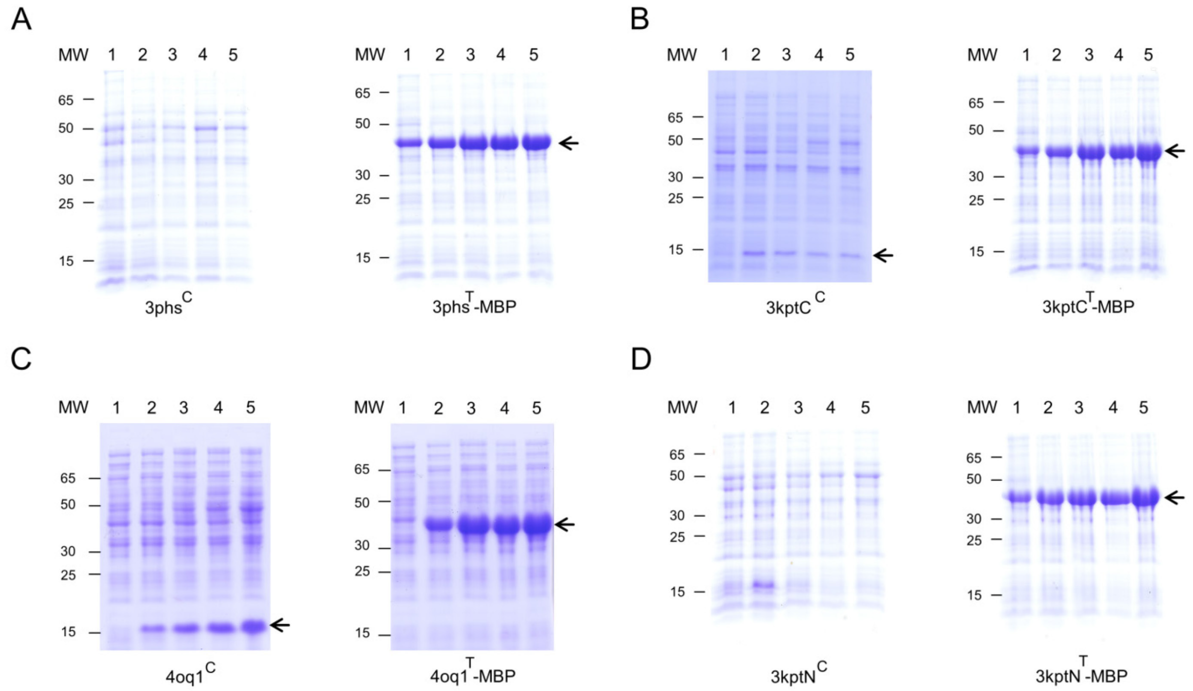

S1 Fig: Time course of heterologous expression of catcher and tag proteins in M15 [pREP4] *E.coli* cells. Expression of recombinant proteins was performed at 28°C with 180 rpm shaking for 4h. Once the cells reached an OD600 of approx. 0.5, expression was induced with 1mM IPTG (0.5mM IPTG for 3kptC^{C}) (final concentration). Recombinant protein expression was monitored by SDS-PAGE and Coomassie Brilliant blue staining of the samples (lane 1: 0h, lane 2: 1h, lane 3: 2h, lane 4: 3h, lane 5: 4h). An arrow indicates the product band. (A) Heterologous expression of 3phs^{C} and 3phs^{T}-MBP, (B) heterologous expression of 3kptC^{C} and 3kptC^{T}-MBP, (C) heterologous expression of 4oq1^{C} and 4oq1^{T}-MBP, (D) heterologous expression of 3kptN^{C} and 3kptN^{T}-MBP. Samples were adjusted to the same OD$_{600}$ and same volume was loaded on the gel. MW stands for molecular weight (kDa).
