# Supplementary material for: Probing the potential of CnaB-type domains for the design of tag/catcher systems
Source: PLoS One. 2017 Jun 27;12(6):e0179740. doi: 10.1371/journal.pone.0179740 (PMC5487036; doi:10.1371/journal.pone.0179740)
Supplement: S2 Fig — (PDF) [file pone.0179740.s002.pdf]

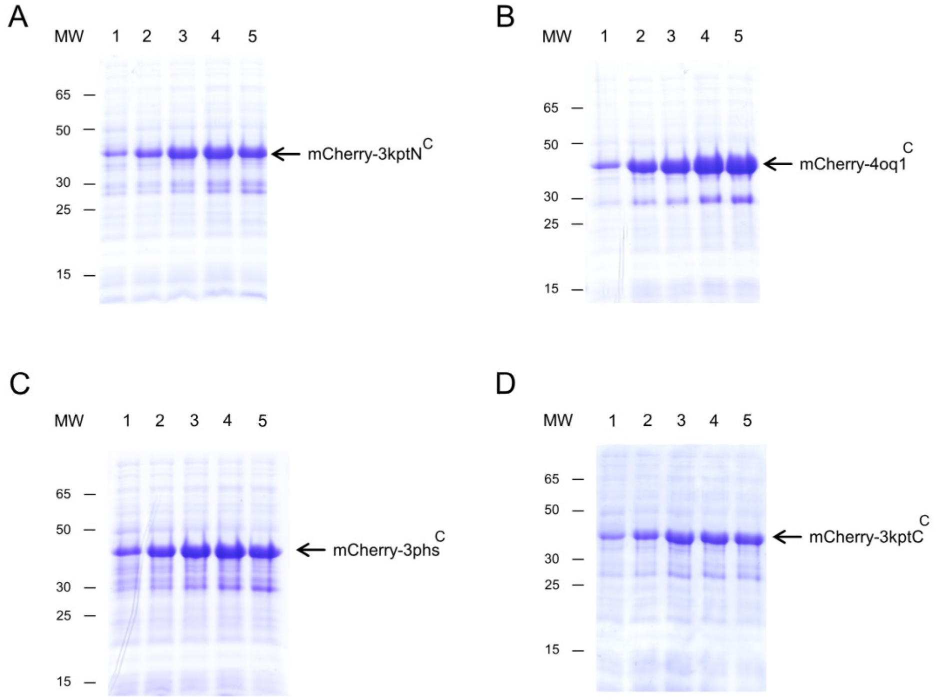

S2 Fig: Time course of heterologous expression of mCherry-catcher fusion proteins in M15 [pREP4] *E.coli* cells. Expression of recombinant proteins was performed at 28°C with 180 rpm shaking for 4h. Once the cells reached an OD_{600} of 0.5 expression was induced with 1mM IPTG (final concentration). Recombinant protein expression was monitored by SDS-PAGE and Coomassie Brilliant blue staining of the samples (lane 1: 0h, lane 2: 1h, lane 3: 2h, lane 4: 3h, lane 5: 4h). (A) mCherry-3kptN^C expression, (B) mCherry-4oq1^C expression, (C) mCherry-3phs^C expression, (D) mCherry-3kptC^C expression. Samples were adjusted to the same OD_{600} and same volume was loaded on the gel. MW stands for molecular weight (kDa).
