# Supplementary material for: Probing the potential of CnaB-type domains for the design of tag/catcher systems
Source: PLoS One. 2017 Jun 27;12(6):e0179740. doi: 10.1371/journal.pone.0179740 (PMC5487036; doi:10.1371/journal.pone.0179740)
Supplement: S3 Fig — (PDF) [file pone.0179740.s003.pdf]

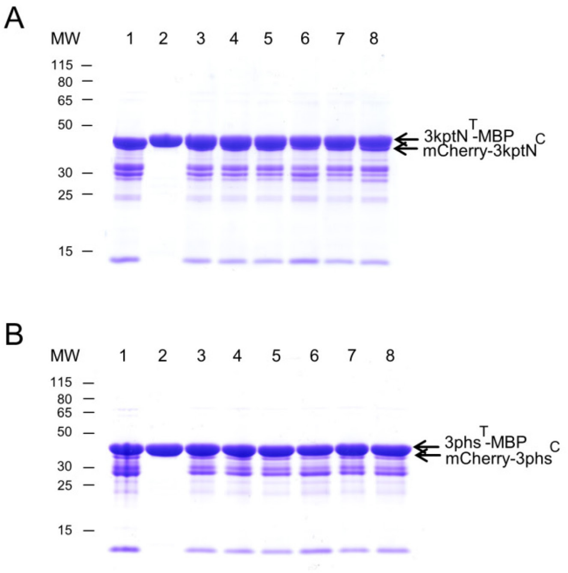

S3 Fig: Covalent intermolecular bond formation assay between tag-MBP and respective mCherry-catcher proteins. Purified tag-MBP and mCherry-catcher proteins were mixed each at 15 μM (final conc.) for 24h at 25°C with shaking at 500 rpm before boiling (10min, 95°C) and SDS-PAGE with Coomassie staining. (A) Time course of 3kptN^T-MBP and mCherry-3kptN^C reaction. (B) Time course of 3phs^T-MBP and mCherry-3phs^C reaction. Lane 1: mCherry-catcher input (30μM), lane 2: tag input (30μM), lane 3: 0h, lane 4: 1h, lane 5: 2h, lane 6: 3h, lane 7: 4h, lane 8: 24h. Same volume was loaded on the gel. MW stands for molecular weight (kDa).
