# Supplementary material for: Probing the potential of CnaB-type domains for the design of tag/catcher systems
Source: PLoS One. 2017 Jun 27;12(6):e0179740. doi: 10.1371/journal.pone.0179740 (PMC5487036; doi:10.1371/journal.pone.0179740)
Supplement: S4 Fig — (PDF) [file pone.0179740.s004.pdf]

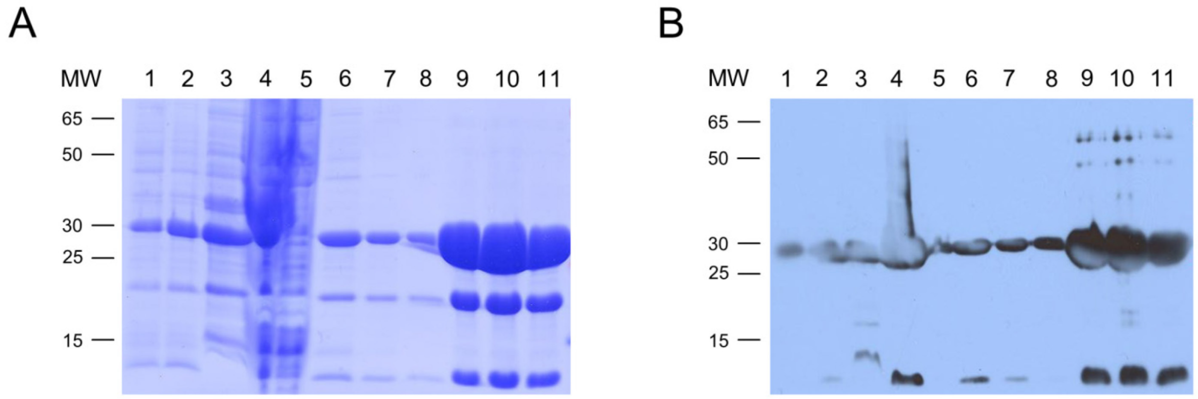

S4 Fig: Purification of recombinantly expressed mCherry$_{only}$ protein via Ni-NTA affinity chromatography. The 6xHis-tagged red fluorescent protein mCherry (pQE-9 expression construct) was expressed in M15 [pREP4] *E.coli* cells and purified using Ni-NTA affinity chromatography under native conditions. Proteins were separated by SDS-PAGE and visualized by Coomassie Brilliant blue staining in A and Anti-His POD Western Blot (1:10000) in B. (A and B: lane 1: non-induced cells → 0h sample of expression time course (OD$_{600}$ adjusted), lane 2: cells induced with IPTG → 4h sample of expression time course (OD$_{600}$ adjusted), lane 3: pellet after cell lysis, lane 4: supernatant after cell lysis, lane 5: flow-through, lane 6-8: 20mM wash, lane 9-11: 250mM elution). Same volume of samples were loaded on Bis-Tris gel after boiling. MW stands for molecular weight (kDa).
