# Supplementary material for: Probing the potential of CnaB-type domains for the design of tag/catcher systems
Source: PLoS One. 2017 Jun 27;12(6):e0179740. doi: 10.1371/journal.pone.0179740 (PMC5487036; doi:10.1371/journal.pone.0179740)
Supplement: S5 Fig — (PDF) [file pone.0179740.s005.pdf]

A

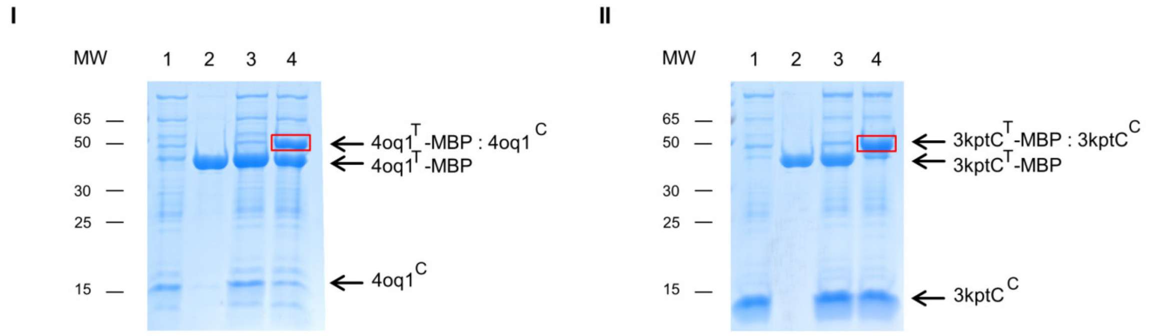

B

I

4oq1^{T}-MBP

MRGSHHHHHHGSVTITVVNQKLPRGNGSGESGKIEEGKLVIWINGDKGYNGLAEVGKKFEKDTGIKVTVEHPDKL
EEKFPQVAATGDGPDIIFWAHDRFGGYAQSGLLAEITPDKAFQDKLYPFTWDAVRYNGKLIAYPIAVEALSLIYNKD
LLPNPPKTWEEIPALDKELKAKGKSALMFNLQEPYFTWPLIAADGGYAFKYENGKYDIKDVGVDNAGAKAGLTFLV
DLIKNKHMNADTDYSIAEAAFNKGETAMTINGPWAWSNIDTSKVNYGVTVLPTFKGQPSKPFVGVLSAGINAASPN
KELAKEFLENYLLTDEGLEAVNKDKPLGAVALKSYEEELAKDPRIAATMENAQKGEIMPNIPQMSAFWYAVRTAVIN
AASGRQTVDEALKDAQTNSSS*

4oq1^{C}

MRGSHHHHHHGSTMTTKVKLIKVDQDHNRLEGVGFKLVSVARDVSEKEVPLIGEYRYSSSGQVGRTLYTDKNGEI
FVTNLPLGNYRFKEVEPLAGYAVTTLDTDVQL*

II

3kptC^{T}-MBP

MRGSHHHHHHGSTVKLTIENNKSPTKGSGESGKIEEGKLVIWINGDKGYNGLAEVGKKFEKDTGIKVTVEHPDKLE
EKFPQVAATGDGPDIIFWAHDRFGGYAQSGLLAEITPDKAFQDKLYPFTWDAVRYNGKLIAYPIAVEALSLIYNKDLL
PNPPKTWEEIPALDKELKAKGKSALMFNLQEPYFTWPLIAADGGYAFKYENGKYDIKDVGVDNAGAKAGLTFLVDL
IKNKHMNADTDYSIAEAAFNKGETAMTINGPWAWSNIDTSKVNYGVTVLPTFKGQPSKPFVGVLSAGINAASPNKE
LAKEFLENYLLTDEGLEAVNKDKPLGAVALKSYEEELAKDPRIAATMENAQKGEIMPNIPQMSAFWYAVRTAVINAA
SGRQTVDEALKDAQTNSSS*

3kptC^{C}

MRGSHHHHHHGSTTGIIELTKIDSANKNKLKGAEFVLKDNNGKIVVVAGKEVTGVSDENGVIKWSNIPYGDYQIFET
KAPTYTKEDGTKTSYQLLKDPIDVKIS*

S5 Fig: Mass spectrometric analysis of 4oq1 and 3kpt product band. (A) Purified catcher (50µM final conc.) and tag-MBP (10µM final conc.) proteins were mixed at 25°C with shaking (500rpm) for 24h prior to boiling (10min, 95°C) and loading on a SDS-gel afterwards stained with brilliant blue colloidal concentrate (Sigma-Aldrich) (lane 1: catcher input (50µM), lane2: tag input (10µM), lane3: 0h sample, lane4: 24h sample). Corresponding product bands were cut out (red box). In-gel trypsin digest and subsequent MS analysis were performed (for further details see material and method). (B) Amino acid coverage of the gel-extracted and trypsin digested product band based on MS identified peptides (for details see S7 Table and S8 Table). Peptides identified by MS analysis are highlighted in green. The reactive amino acids are marked red.
