# Supplementary material for: Probing the potential of CnaB-type domains for the design of tag/catcher systems
Source: PLoS One. 2017 Jun 27;12(6):e0179740. doi: 10.1371/journal.pone.0179740 (PMC5487036; doi:10.1371/journal.pone.0179740)
Supplement: S6 Fig — (PDF) [file pone.0179740.s006.pdf]

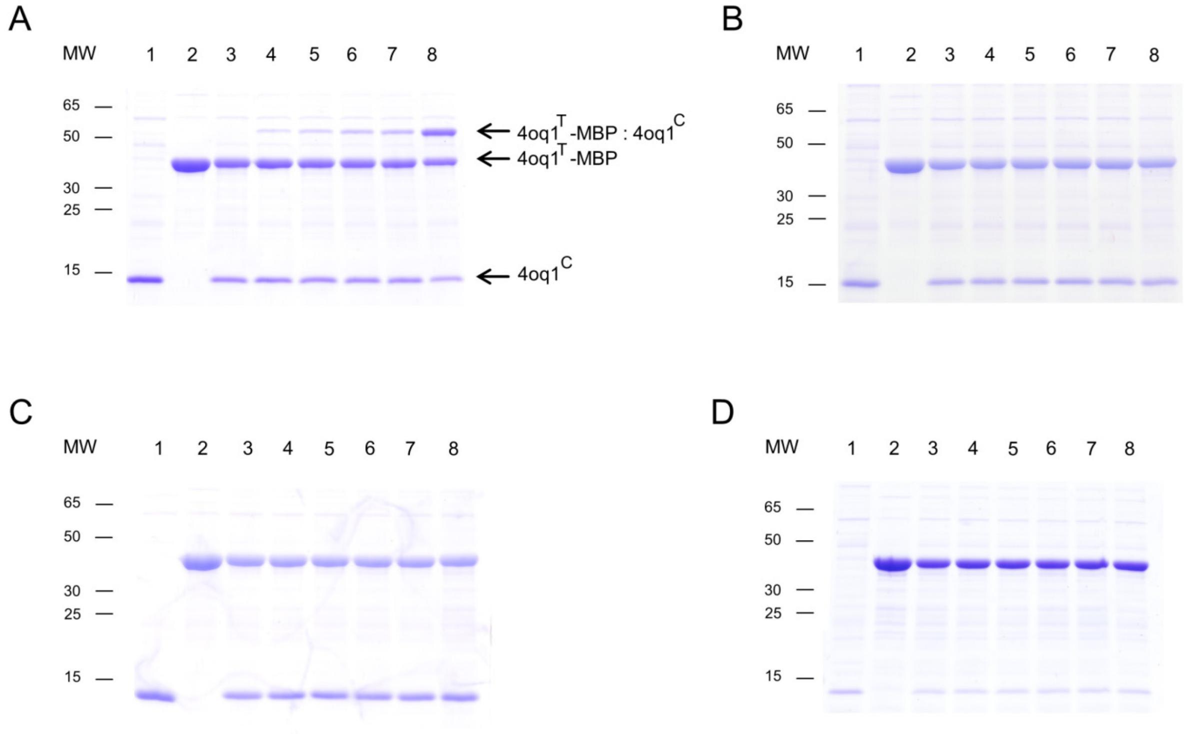

S6 Fig: Covalent bond formation assays of 4oq1 active site mutants.
(A) 4oq1^C+4oq1^T-MBP (wildtype interaction); (B) 4oq1^C + 4oq1^T(N252A)-MBP; (C) 4oq1^C(K155A) + 4oq1^T-MBP; (D) 4oq1^C(E222Q) + 4oq1^T-MBP. The wildtype interaction showed a covalent product increasing over time whereas no covalent product was detected in the active site mutants. Comparative covalent bond formation assays were performed with 15µM final concentration of the reactants. The reactive residues N252 in the 4oq1^T and K155 and E222 in the 4oq1^C were mutated and tested for covalent product formation via SDS-PAGE and subsequent Coomassie Brilliant blue staining. Samples were boiled at 95°C for 10min prior to gel loading. (A-D): lane 1: catcher input (30µM), lane 2: tag input (30µM), lane 3: 0h, lane 4: 1h, lane 5: 2h, lane 6: 3h, lane 7: 4h, lane 8: 24h). Same volume of samples were loaded. MW stands for molecular weight (kDa).
