# Supplementary material for: Probing the potential of CnaB-type domains for the design of tag/catcher systems
Source: PLoS One. 2017 Jun 27;12(6):e0179740. doi: 10.1371/journal.pone.0179740 (PMC5487036; doi:10.1371/journal.pone.0179740)
Supplement: S8 Fig — (PDF) [file pone.0179740.s008.pdf]

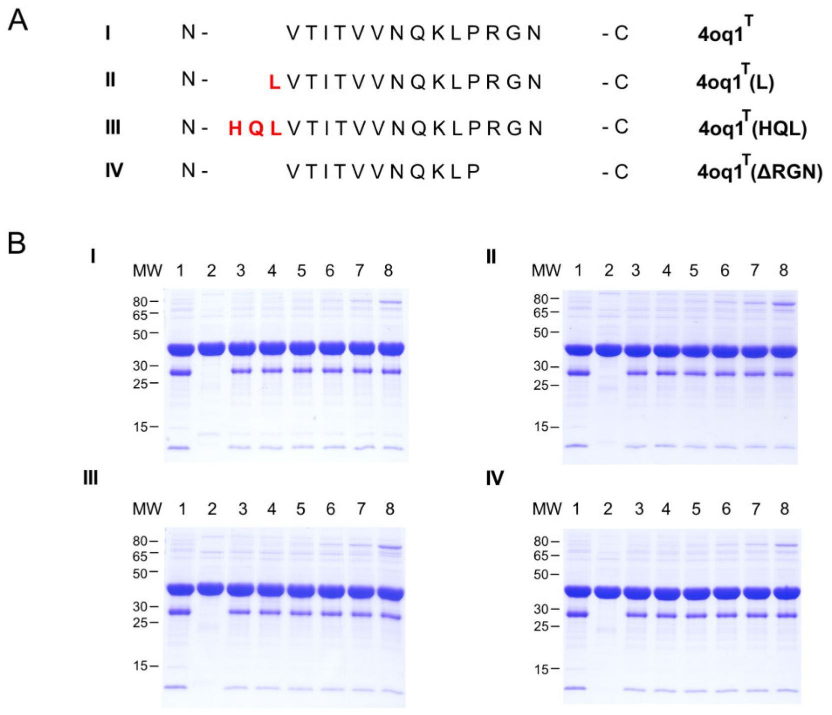

S8 Fig: Rates of isopeptide bond formation of 4oq1^T-MBP variants on the minute scale. (A) Sequence alignment of different 4oq1^T-MBP variants (I: 4oq1^T wildtype (residues V246-N259), II: N-terminal L245 extension of 4oq1^T, III: N-terminal H243-Q244-L245 extension of 4oq1^T, IV: C-terminal R257-G258-N259 truncation of 4oq1^T). (B) Comparative covalent intermolecular bond formation assay between different 4oq1^T-MBP variants and mCherry-4oq1^C (0min-30min). Purified 4oq1^T-MBP variants and mCherry-4oq1^C were mixed each at 15 µM (final conc.) for 30min at 25°C with shaking at 500 rpm before boiling (10min, 95°C) and SDS-PAGE with Coomassie staining. Interaction I: mCherry-4oq1^C + 4oq1^T (wildtype), interaction II: mCherry-4oq1^C + 4oq1^T (L), interaction III: mCherry-4oq1^C + 4oq1^T (HQL), interaction IV: mCherry-4oq1^C + 4oq1^T (ΔRGN). (lane 1: mCherry-catcher input (30µM), lane 2: tag input (30µM), lane 3: 0min, lane 4: 1min, lane 5: 3min, lane 6: 5min, lane 7: 10min, lane 8: 30min). Same volume was loaded on the gel. MW stands for molecular weight (kDa).
