# Supplementary material for: Probing the potential of CnaB-type domains for the design of tag/catcher systems
Source: PLoS One. 2017 Jun 27;12(6):e0179740. doi: 10.1371/journal.pone.0179740 (PMC5487036; doi:10.1371/journal.pone.0179740)
Supplement: S9 Fig — (PDF) [file pone.0179740.s009.pdf]

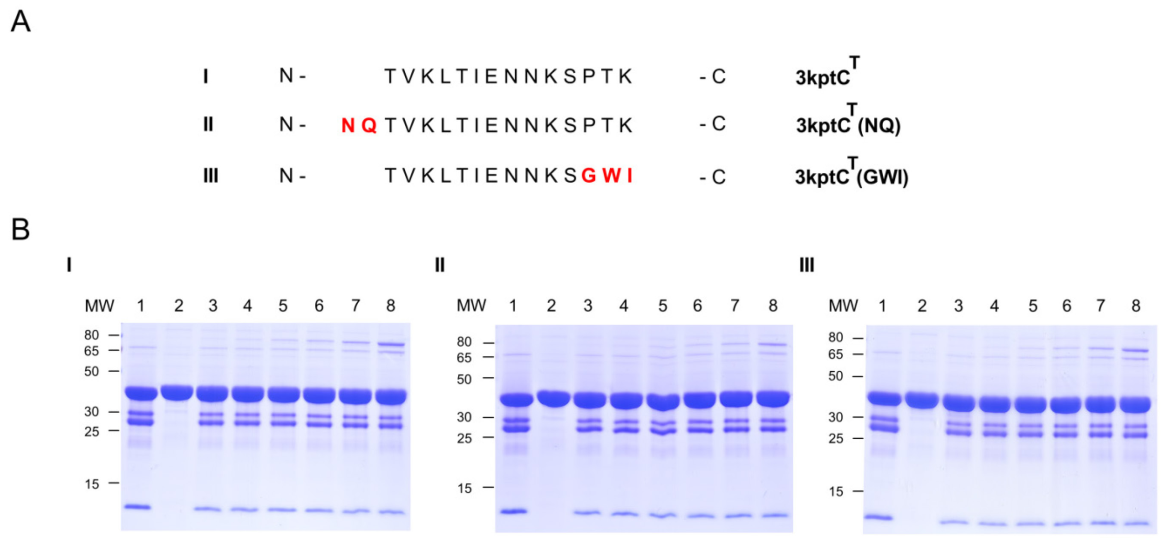

S9 Fig: Rates of isopeptide bond formation of 3kptC^{T}-MBP variants on the minute scale.

(A) Sequence alignment of different 3kptC^{T}-MBP variants (I: 3kptC^{T} wildtype (residues T505-K518), II: N-terminal N506-Q507 extension of 3kptC^{T}, III: C-terminal GWI instead of PTK in 3kptC^{T}). (B) Comparative covalent intermolecular bond formation assay between different 3kptC^{T}-MBP variants and mCherry-3kptC^{C} (0min-30min). Purified 3kptC^{T}-MBP variants and mCherry-3kptC^{C} proteins were mixed each at 15 μM (final conc.) for 30min at 25°C with shaking at 500 rpm before boiling (10min, 95°C) and SDS-PAGE with Coomassie staining. Interaction I: mCherry-3kptC^{C} + 3kptC^{T} (wildtype), interaction II: mCherry-3kptC^{C} + 3kptC^{T} (NQ), interaction III: mCherry-3kptC^{C} + 3kptC^{T} (GWI). (lane 1: mCherry-catcher input (30μM), lane 2: tag input (30μM), lane 3: 0min, lane 4: 1min, lane 5: 3min, lane 6: 5min, lane 7: 10min, lane 8: 30min). Same volume was loaded on the gel. MW stands for molecular weight (kDa).
