# Supplementary material for: Probing the potential of CnaB-type domains for the design of tag/catcher systems
Source: PLoS One. 2017 Jun 27;12(6):e0179740. doi: 10.1371/journal.pone.0179740 (PMC5487036; doi:10.1371/journal.pone.0179740)
Supplement: S10 Fig — (PDF) [file pone.0179740.s010.pdf]

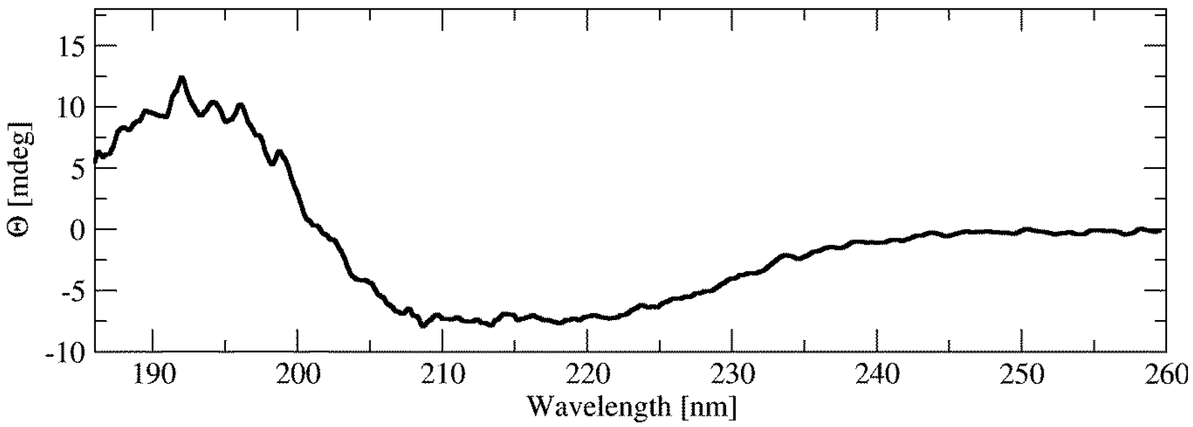

S10 Fig: Far-UV CD-spectrum of 4oq1[C]. The CD spectrum was measured at 20°C over the wavelength range of 185-260 nm. 4oq1[C] protein was used in a concentration of 0.3 mg/ml in 10mM sodium phosphate buffer pH 7. Per measured sample six scans were acquired and accumulated afterwards.
