# Supplementary material for: Probing the potential of CnaB-type domains for the design of tag/catcher systems
Source: PLoS One. 2017 Jun 27;12(6):e0179740. doi: 10.1371/journal.pone.0179740 (PMC5487036; doi:10.1371/journal.pone.0179740)
Supplement: S1 Table — (PDF) [file pone.0179740.s011.pdf]

S1 Table: Primers used in the present study

1	5´ - CCTGGGATCCGTGAGCAAGGGCGAGGAGGATAAC - 3´
2	5´ - GGATCTGGTTCAGGCTCCGGAGAAATTAAACGTGGCGCGGTG - 3´
3	5´ - CACCGCGCCACGTTTAATTTCTCCGGAGCCTGAACCAGATCC - 3´
4	5´ - CAGGGTCGACTTAAGAGGTAATGGTGCCATCTTC - 3´
5	5´ - GGATCTGGTTCAGGCTCCGGAACCATGACCACCAAAGTGAAAC - 3´
6	5´ - GTTTCACTTTGGTGGTCATGGTTCCGGAGCCTGAACCAGATCC - 3´
7	5´ - CAGGGTCGACTTACAGCTGCACATCGGTATCCAG - 3´
8	5´ - GGATCTGGTTCAGGCTCCGGATCTACCGGCGAATTAGATTTAC - 3´
9	5´ - GTAAATCTAATTCGCCGGTAGATCCGGAGCCTGAACCAGATCC - 3´
10	5´ - CAGGGTCGACTTATTCAATGGTCACCGCGGTTTC - 3´
11	5´ - GGATCTGGTTCAGGCTCCGGAACCACCGGCATTATTGAATTAAC - 3`
12	5´ - GTTAATTCAATAATGCCGGTGGTTCCGGAGCCTGAACCAGATCC - 3´
13	5´ - CAGGGTCGACTTAGCTAATTTTCACATCAATCG - 3´
14	5´ - CCTGGGATCCGTGACCATTACCGTGGTGAACCAGAAATTACCGCGTGGCAA CGGTAGTGGTGAAAGTGGTAAAATCGAAGAAGGTAAACTGG - 3´
15	5´ - CAGGGTCGACTTACGAGCTCGAATTAGTCTGCGCGTCTTTCAGGGCTTCATC - 3´
16	5´ - CCTGGGATCCCATCAGCTGGTGACCATTACCGTGGTGAACCAG - 3´
17	5´ - CCTGGGATCCCTGGTGACCATTACCGTGGTGAACC - 3´
18	5´ - CCTGGGATCCGTGACCATTACCGTGGTGAACCAGAAATTACCGGGTAGTGG TGAAAGTGGTAAAATC - 3´
19	5´ - CCTGGGATCCACCGTGAAATTAACCATTGAAAACAACAAAAGCCCGACC AAAGGTAGTGGTGAAAGTGGTAAAATCG - 3`
20	5´ - CCTGGGATCCAACCAGACCGTGAAATTAACCATTGAAAAC - 3`
21	5´ - CCTGGGATCCACCGTGAAATTAACCATTGAAAACAACAAAAGCGGCTGG ATTGGTAGTGGTGAAAGTGGTAAAATC- 3´
22	5´ - CCTGGGATCCGTGACCATTACCGTGGTGGCGCAGAAATTACCGCGTGGCAAC - 3`
23	5´ - CAGGGTCGACTTACGAGCTCGAATTAGTCTGCGCGTCTTTCAGGGCTTCATC - 3`
24	5´ - CCTGGGATCCACCATGACCACCAAAGTGAAACTGATTGCGGTGGATCAGGATCATAA CCG - 3`
25	5´ - CAGGGTCGACTTACAGCTGCACATCGGTATCCAG - 3`
26	5´ - CCTGGGATCCACCATGACCACC - 3`

27	5´ - CAGGGTCGACTTACAGCTGCACATCGGTATCCAGGGTGGTCACCGCATAGC CCGCCAGCGGTTCCACCTGTTTAAAACG - 3`
28	5´ - CCTGGGATCCACCGTGAAATTAACCATTGAAGCGAACAAAAGCCCGACCAAAGGTAG - 3`
29	5´ - CAGGGTCGACTTACGAGCTCGAATTAGTCTGCGCGTCTTTCAGGGCTTCATC - 3`
30	5´ - CCTGGGATCCACCACCGGCATTATTGAATTAACCGCGATTGATAGCGCG AACAAAAAC - 3´
31	5´ - CAGGGTCGACTTAGCTAATTTTCACATCAATCG - 3`
32	5´ - GTATGGCGATTATCAGATTTTTCAGACCAAAGCGCCGACCTATAC - 3´
33	5´ - GTTTCGCCACCTCTGACTTGAG - 3´
34	5´ - CTCAAGTCAGAGGTGGCGAAAC - 3´
35	5´ - GTATAGGTCGGCGCTTTGGTCTGAAAAATCTGATAATCGCCATAC -3´
