# Supplementary material for: Probing the potential of CnaB-type domains for the design of tag/catcher systems
Source: PLoS One. 2017 Jun 27;12(6):e0179740. doi: 10.1371/journal.pone.0179740 (PMC5487036; doi:10.1371/journal.pone.0179740)
Supplement: S2 Table — (PDF) [file pone.0179740.s012.pdf]

S2 Table: Cloning scheme for mCherry-GSGSGSG-Catcher constructs

**mCherry- -3kptC^{C}**	**mCherry --3kptN^{C}**	**mCherry--3phs^{C}**	**mCherry- -4oq1^{C}**
PCR1:<br>1 + 12;<br>mCherry as template	PCR1:<br>1 + 3;<br>mCherry as template	PCR1:<br>1 + 9;<br>mCherry as template	PCR1:<br>1 + 6;<br>mCherry as template
PCR2:<br>11 + 13;<br>Synthetic gene as template	PCR2:<br>2 + 4;<br>Synthetic gene as template	PCR2:<br>8 + 10;<br>Synthetic gene as template	PCR2:<br>5 + 7;<br>Synthetic gene as template
Overlap PCR:<br>1 +13<br>Products from PCR1 and PCR2 as templates	Overlap PCR:<br>1 + 4<br>Products from PCR1 and PCR2 as templates	Overlap PCR:<br>1 + 10<br>Products from PCR1 and PCR2 as templates	Overlap PCR:<br>1 + 7<br>Products from PCR1 and PCR2 as templates

Number of primers used correlate with the primer list in S1 Table.
