# Supplementary material for: Probing the potential of CnaB-type domains for the design of tag/catcher systems
Source: PLoS One. 2017 Jun 27;12(6):e0179740. doi: 10.1371/journal.pone.0179740 (PMC5487036; doi:10.1371/journal.pone.0179740)
Supplement: S3 Table — (PDF) [file pone.0179740.s013.pdf]

S3 Table: Cloning scheme for 4oq1^T-MBP variants (GSGESG linker and MBP sequence from pMAL-c2 vector)

**4oq1^T(wildtype)**	**4oq1^T(L)**	**4oq1^T(HQL)**	**4oq1^T(ΔRGN)**
PCR: 14 + 15 4oq1^T-GSGSGSG-MBP as template	PCR: 17 + 15 4oq1^T(wildtype) as template	PCR: 16 + 15 4oq1^T(wildtype) as template	PCR: 18 + 15 4oq1^T(wildtype) as template

Number of primers used correlate with the primer list in S1 Table.
