# Supplementary material for: Probing the potential of CnaB-type domains for the design of tag/catcher systems
Source: PLoS One. 2017 Jun 27;12(6):e0179740. doi: 10.1371/journal.pone.0179740 (PMC5487036; doi:10.1371/journal.pone.0179740)
Supplement: S4 Table — (PDF) [file pone.0179740.s014.pdf]

S4 Table: Cloning scheme for 3kptC^T-MBP variants (GSGESG linker and MBP sequence from pMAL-c2 vector)

**3kptC^T(wildtype)**	**3kptC^T(NQ)**	**3kptC^T(GWI)**
PCR: 19 + 15<br>3kptC^T-GSGSGSG-MBP as template	PCR: 20 + 15<br>3kptC^T(wildtype) as template	PCR: 21 + 15<br>3kptC^T(wildtype) as template

Number of primers used correlate with the primer list in S1 Table.
