# Supplementary material for: Probing the potential of CnaB-type domains for the design of tag/catcher systems
Source: PLoS One. 2017 Jun 27;12(6):e0179740. doi: 10.1371/journal.pone.0179740 (PMC5487036; doi:10.1371/journal.pone.0179740)
Supplement: S5 Table — (PDF) [file pone.0179740.s015.pdf]

S5 Table: Cloning scheme for non-reactive 4oq1 variants (GSGESG linker and MBP sequence from pMAL-c2 vector)

**4oq1^{T}(N252A)**	**4oq1^{C}(K155A)**	**4oq1^{C}(E222Q)**
PCR: 22 + 23<br>4oq1^{T}-GSGESG-MBP as template	PCR: 24 + 25<br>4oq1^{C} as template	PCR: 26 + 27<br>4oq1^{C} as template

Number of primers used correlate with the primer list in S1 Table.
