# Supplementary material for: Probing the potential of CnaB-type domains for the design of tag/catcher systems
Source: PLoS One. 2017 Jun 27;12(6):e0179740. doi: 10.1371/journal.pone.0179740 (PMC5487036; doi:10.1371/journal.pone.0179740)
Supplement: S6 Table — (PDF) [file pone.0179740.s016.pdf]

S6 Table: Cloning scheme for non-reactive 3kptC variants (GSGESG linker and MBP sequence from pMAL-c2 vector)

**3kptC^T(N512A)**	**3kptC^C(K417A)**	**3kptC^C(E472Q)**
PCR: 28 + 29<br>3kptC^T-GSGESG-MBP as template	PCR: 30 + 31<br>3kptC^C as template	PCR1: 32 + 33<br>PCR2: 34 + 35<br>3kptC^C in pQE-9 vector as template

Number of primers used correlate with the primer list in S1 Table.
